# End-to-end sequence-structure-function meta-learning predicts genome-wide chemical-protein interactions for dark proteins

**DOI:** 10.1371/journal.pcbi.1010851

**Published:** 2023-01-18

**Authors:** Tian Cai, Li Xie, Shuo Zhang, Muge Chen, Di He, Amitesh Badkul, Yang Liu, Hari Krishna Namballa, Michael Dorogan, Wayne W. Harding, Cameron Mura, Philip E. Bourne, Lei Xie

**Affiliations:** 1 Ph.D. Program in Computer Science, The Graduate Center, The City University of New York, New York, New York, United States of America; 2 Department of Computer Science, Hunter College, The City University of New York, New York, New York, United States of America; 3 Master Program in Computer Science, Courant Institute of Mathematical Sciences, New York University, New York, New York, United States of America; 4 Department of Chemistry, Hunter College, The City University of New York, New York, New York, United States of America; 5 School of Data Science & Department of Biomedical Engineering, University of Virginia, Charlottesville, Virginia, United States of America; 6 Helen and Robert Appel Alzheimer’s Disease Research Institute, Feil Family Brain & Mind Research Institute, Weill Cornell Medicine, Cornell University, New York, New York, United States of America; Georgia Institute of Technology, UNITED STATES

## Abstract

Systematically discovering protein-ligand interactions across the entire human and pathogen genomes is critical in chemical genomics, protein function prediction, drug discovery, and many other areas. However, more than 90% of gene families remain “dark”—i.e., their small-molecule ligands are undiscovered due to experimental limitations or human/historical biases. Existing computational approaches typically fail when the dark protein differs from those with known ligands. To address this challenge, we have developed a deep learning framework, called PortalCG, which consists of four novel components: (i) a 3-dimensional ligand binding site enhanced sequence pre-training strategy to encode the evolutionary links between ligand-binding sites across gene families; (ii) an end-to-end pretraining-fine-tuning strategy to reduce the impact of inaccuracy of predicted structures on function predictions by recognizing the sequence-structure-function paradigm; (iii) a new out-of-cluster meta-learning algorithm that extracts and accumulates information learned from predicting ligands of distinct gene families (meta-data) and applies the meta-data to a dark gene family; and (iv) a stress model selection step, using different gene families in the test data from those in the training and development data sets to facilitate model deployment in a real-world scenario. In extensive and rigorous benchmark experiments, PortalCG considerably outperformed state-of-the-art techniques of machine learning and protein-ligand docking when applied to dark gene families, and demonstrated its generalization power for target identifications and compound screenings under out-of-distribution (OOD) scenarios. Furthermore, in an external validation for the multi-target compound screening, the performance of PortalCG surpassed the rational design from medicinal chemists. Our results also suggest that a differentiable sequence-structure-function deep learning framework, where protein structural information serves as an intermediate layer, could be superior to conventional methodology where predicted protein structures were used for the compound screening. We applied PortalCG to two case studies to exemplify its potential in drug discovery: designing selective dual-antagonists of dopamine receptors for the treatment of opioid use disorder (OUD), and illuminating the understudied human genome for target diseases that do not yet have effective and safe therapeutics. Our results suggested that PortalCG is a viable solution to the OOD problem in exploring understudied regions of protein functional space.

## Author summary

Many complex diseases, such as Alzheimer’s disease, mental disorders, and substance use disorders, do not have safe and effective therapeutics because of the polygenic nature of the diseases and a lack of thoroughly validated drug targets (and their corresponding ligands). Identifying small-molecule ligands for all proteins encoded in the human genome would provide powerful new opportunities for drug discovery of currently untreatable diseases. However, the small-molecule ligand of more than 90% of gene families is completely unknown. Existing protein-ligand docking and machine learning methods often fail when the protein of interest is dissimilar to those with known functions or structures. We have developed a new deep learning framework, PortalCG, for efficiently and accurately predicting ligands of understudied proteins which are out of reach of existing methods. Our method achieves unprecedented accuracy versus state-of-the-art approaches, and it achieves this by incorporating ligand binding site information and the sequence-to-structure-to-function paradigm into a novel deep meta-learning algorithm. In a case study, the performance of PortalCG surpassed the rational design from medicinal chemists. The proposed computational framework can shed new light on how chemicals modulate biological systems, which is indispensable in drug repurposing and rational design of polypharmacology. This approach could offer a new way to develop safe and effective therapeutics for currently incurable diseases. PortalCG can be extended to other types of tasks, such as predicting protein-protein interactions and protein-nucleic acid recognition.

## Introduction

Scientific inquiry always aims to deduce new concepts from existing knowledge or to generalize observations, and numerous such challenges and opportunities exist in the biological sciences. The rise of deep learning has sparked a surge of interest in using machine learning to explore previously uncharted molecular and functional spaces in biology and medicine, ranging from “deorphanizing” G-protein coupled receptors [1] and translating cell-line screens to patient drug responses [2, 3], to predicting novel protein structures [4–6], to identifying new cell types from single-cell omics data [7]. Illuminating the understudied space of human knowledge is a fundamental problem that one can attempt to address via deep learning—that is, to generalize a “well-trained” model to unseen data that lies Out-of-Distribution (OOD) of the training data, in order to successfully predict outcomes under conditions that the model has never encountered before. While deep learning is capable, in theory, of simulating any function mapping, its generalization power is notoriously limited in the case of distribution shifts [8].

The training of a deep learning model starts with a domain-specific model architecture. The final model instance that is selected for deployment, and its performance, are determined by a series of data-dependent design choices, including model initialization, how data are split and used for training/validation/testing sets, optimization of loss function, and evaluation metrics. Each of these design choices impacts the generalization power of a trained model. The development of several recent deep learning-based approaches—notably transfer learning [9], self-supervised representation learning [10], and meta-learning [11, 12]—has been motivated by the OOD challenge. However, each of these approaches focuses on only one aspect in the training pipeline of a deep neural network model. Causal learning and mechanism-based modeling (e.g., based on physical first principles) could be an effective way to circumvent the OOD problem [8], but at present these approaches can be applied only on modest scales because of data scarcity, computational complexity, or limited domain knowledge. Solving large-scale OOD problems in biomedicine via machine learning would benefit from a systematic framework for integrative, end-to-end model development and deployment, as well as the incorporation of domain knowledge into the training process.

OOD challenges are commonplace in drug discovery and development because of the vastness of chemical genomics space: small molecules act as endogenous or exogenous ligands of numerous proteins, assisting in maintaining homeostasis of a biological system or serving as therapeutics agents to alter pathological processes. Despite tremendous progress in high-throughput screening, the majority of protein space remains unexplored [13] due to high costs, inherent limitations in experimental approaches, and human biases [14, 15]. Even in well-studied gene families, such as G-protein coupled receptors (GPCRs), protein kinases, ion channels, and estrogen receptors, a large portion of proteins remain dark [13], i.e., their ligands remain unknown. Elucidating the ligand-binding properties of dark proteins and gene families can shed light on many essential but poorly understood biological processes, such as microbiome-host interactions mediated by metabolite-protein interactions. Such efforts could also be instrumental for drug discovery. Firstly, although the conventional one-drug-one-gene drug discovery process focuses on screening drugs against a single target, unrecognized off-target effects are a common occurrence [16]. The off-target effects can either be the cause of undesirable side effects or present a unique potential opportunity for drug repurposing. Secondly, polypharmacology—i.e., designing drugs that can target multiple proteins—is needed to achieve desired therapeutic efficacy and combat drug resistance for multi-genic diseases [16]. Finally, identifying new druggable targets and discovering their ligands may provide effective therapeutic strategies for currently incurable diseases; for instance, in Alzheimer’s disease (AD), many disease-associated genes have been identified through multiple omics studies, but are presently considered as dark proteins [17].

Accurate and robust prediction of chemical-protein interactions (CPIs) across the genome is a challenging OOD problem [1]. If one considers only the reported area under the receiver operating characteristic curve (AUROC), which has achieved values as high as 0.9 in many state-of-the-art methods [18, 19], it may seem that the problem has been solved. However, existing methods have rarely been applied to dark gene families. The performance of existing methods has been assessed primarily in scenarios where the data distribution in the test set does not differ significantly from that in the training set, in terms of similarities between proteins or between chemicals; that is, the development of current methods involved sampling quite limited regions of protein space. Few sequence-based methods have been developed and evaluated for an out–of–gene-family scenario, where proteins in the test set belong to different (non-homologous) gene families from those in the training set; this sampling bias is even more severe in considering cases where the new gene family does not have any reliable three-dimensional (3D) structural information. Therefore, one can fairly claim that all existing machine learning work has been confined to just narrow regions of chemical genomics space for an imputation task, without validated generalizability into the dark proteins for novel discoveries. With the advent of high-accuracy protein structural models, predicted by AlphaFold2 [5], it now becomes possible to use reversed protein-ligand docking (PLD) [20] to predict ligand-binding sites and poses on dark proteins on a genome-wide scale. However, AlphaFold2 can only provide structural models for around half of dark human proteins [21]. Furthermore, it is well known that PLD suffers from a high false-positive rate due to poor modeling of protein conformational dynamics, solvation effects, crystallized waters, and other challenges [22]; for example, small-molecule ligands will often be found to indiscriminately “stick” to concave, pocket-like patches on protein surfaces. For these reasons, the relatively low reliability of PLD still poses a significant limitation [23]. Thus, the direct application of PLD remains a challenge and a limited scope for predicting ligand binding to dark proteins.

In this paper, we propose a new deep learning framework, “Portal Learning”, and its application to chemical genomics (“PortalCG”), for predicting small-molecule binding to “dark” proteins (whose ligands are unknown) and to dark gene families (wherein all protein members do not have known ligands). Here, we use the word “Portal” to represent multiple training components in an end-to-end deep learning framework, structured so as to be able to systematically address OOD challenges. We show that PortalCG significantly outperforms the leading machine learning and protein-ligand docking methods that are available for predicting ligand binding to dark proteins. Thus, PortalCG may shed new light on unknown functions for dark proteins, and empower drug discovery using Artificial Intelligence (AI). To demonstrate the potential of PortalCG, this work applies it to two case studies: (i) designing selective dual-antagonists of Dopamine receptors for Opioid Use Disorder (OUD) with experimental validations, and (ii) illuminating the understudied druggable genome for targeting diseases that lack effective and safe therapeutics. The novel genes and their lead compounds identified from PortalCG provide new opportunities for drug discovery to treat currently incurable diseases, such as OUDs and AD. We believe that these predictions warrant further experimental validation and exploration.

In summary, the contributions of this work are two-fold:

1. We develop and test a new algorithm, PortalCG, to improve the generalization power of machine learning on OOD problems. Comprehensive benchmark studies demonstrate the promise of PortalCG when applied to exploring dark gene families (i.e., those consisting of proteins with no known small-molecule ligands)
2. Using PortalCG, we shed new light on unknown protein functions in dark proteins (viz. small molecule-binding properties), and open new avenues in polypharmacology and drug repurposing; the latter is demonstrated by our identification of novel drug targets and lead compounds for OUDs and AD

## Results and discusssion

**Fig 1:**
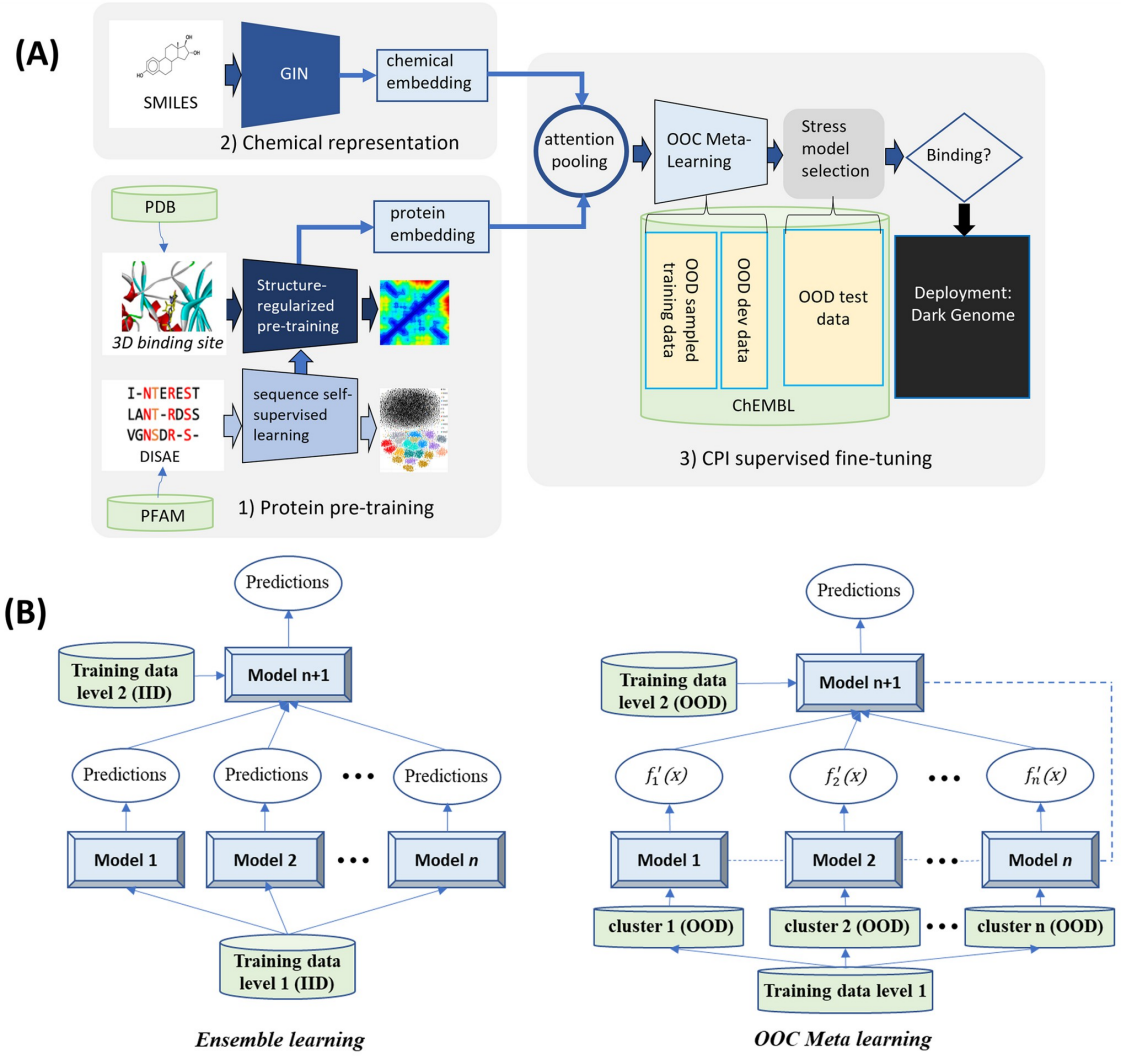
(A) Design Scheme of PortalCG: PortalCG enables the prediction of chemical-protein interactions (CPIs) for dark proteins, across gene families, via four key components: (i) ligand-binding site enhanced sequence pretraining, (ii) end-to-end transfer learning, in accord with the sequence-structure-function paradigm, (iii) out-of-cluster meta-learning (OOC-ML), and (iv) stress model selection. (B) How OOC-ML compares to classic stacking ensemble learning: OOC-ML is similar in spirit to stacking ensemble learning, but differs in data split strategies, model architecture, and optimization schema, as further detailed in the text.

PortalCG includes four key, biology-inspired components, as schematized in Fig 1: 3-dimensional (3D) binding site-enhanced sequence pre-training, end-to-end sequence-structure-function step-wise transfer learning (STL), out-of-cluster meta-learning (OOC-ML), and stress model selection. We now describe these model components in turn.

### 3D binding site-enhanced sequence pre-training

Pre-training strategy is a proven powerful approach to boost the generalizability of deep learning models [24]. Pre-trained natural language models have revolutionized Natural Language Processing (NLP) [24]. Significant improvements are also observed when applying the same pre-training strategy to protein sequences for structure [5, 25], function [26, 27], and CPI predictions [1]. We begin by performing self-supervised training to map tens of millions of sequences into a universal embedding space, using a state-of-the-art *distilled sequence alignment embedding* (DISAE) algorithm [1]. In brief, DISAE first distills the original sequence into an ordered list of amino acid triplets by extracting evolutionarily important positions from a multiple sequence alignment. Then, long-range residue-residue interactions can be learned via the Transformer module in ALBERT [10]. A self-supervised masked language modeling (MLM) approach is used to train the model, where 15% triplets are randomly masked and assumed as unknown. The remaining triplets are used to predict what the masked triplets are. In this way, DISAE can learn protein sequence representations that capture functional information without explicit knowledge of (exposure to) either structural or functional data.

3D structural information about ligand-binding sites was used to fine-tune the sequence embedding because such information (i) is sensitive to evolutionarily relationships across fold space and (ii) is more informative than the sequence alone for ligand-binding [28]. In addition to the self-supervised MLM task, amino acid residue-ligand atom distance matrices that were generated from protein-ligand complex structures were predicted from the distilled amino acid triplets. As a result, the original DISAE embedding could be refined with this 3D ligand-binding site information. This structure-regularized protein embedding was used as a hidden layer for supervised learning of cross–gene-family CPIs, following an end-to-end sequence-structure-function training process described below.

### End-to-end, sequence-structure-function–based step-wise transfer learning (STL)

The function of a protein (e.g., serving as a target receptor for ligand binding) stems from its 3D shape and conformational dynamics which, in turn, is ultimately encoded in its primary amino acid sequence. In general, information about a protein’s structure is more powerful than purely sequence-based information for predicting its molecular function because sequences drift/diverge far more rapidly than do 3D structures on evolutionary timescales. Furthermore, proteins from different gene families may have similar functional sites through the convergent evolution, thus perform similar functions [28]. Although the number of experimentally-determined structures continues to exponentially increase—and now AlphaFold2 can reliably predict the 3D structure of a generic single-domain protein—it nevertheless remains quite challenging to directly use protein structures as input for predicting ligand-binding properties of dark proteins. This motivates us to directly use protein sequences to predict ligands of dark proteins in PortalCG. Protein structure information is used as an intermediate layer, as trained by the structure-enhanced pre-training, to connect a protein sequence and a corresponding protein function (Fig 1A), as inspired by the concept of “differentiable biology” [29]. By encapsulating the role of structure in this way, inaccuracies and uncertainties in structure prediction are “insulated” and will not propagate to the function prediction. Details of neural network architecture and training methods can be found in section Algorithm.

### Out-of-cluster meta-learning (OOC-ML)

We designed a new OOC-ML approach to explore dark gene families. Here, predicting ligands of dark gene families can be formulated as the following problem: how can we quickly learn the ligand-binding pattern of a new gene family, lacking any labeled data, from the information obtained from other, well-characterized gene families (that themselves enjoy a relatively large amount of labeled data)? Meta-learning is a general learning strategy that learns a new task without any (or with very few) labeled data from outputs (meta-data) generated by multiple other tasks with labeled data; thus, this approach naturally fits our purpose. The principle of OOC-ML is first to independently learn the pattern of ligand bindings from each gene family that has labeled data, and then to extract the common intrinsic pattern shared by these gene families and apply the learned essential knowledge to dark ones. OOC-ML is similar to stacking ensemble learning that uses a machine learning model at a high level (the second level) to learn how to best combine the predictions from other machine learning models at a low level (the first level), as shown in Fig 1B. Nevertheless, there are three key differences between our proposed OOC-ML approach and classic ensemble learning. First, all low-level models in ensemble learning use the same training data, and the training data used in the high-level has the same distribution as that used in the low-level. In the OOC-ML, the training data for each low-level model has a different distribution. Specifically, they come from different Pfam families. The training data in the high-level also uses Pfam families that are different from all others used in the low-level. Second, instead of using different machine learning algorithms in the low-level ensemble model, the model architecture for all models in the OOC meta-learning is the same, as inspired by an approach called Model Agnostic Meta-Learning (MAML) [11]. The difference between models lies in their different parameters (mapping functions) due to the different input data. Finally, ensemble learning uses the predictions from the low-level models as meta-data for the input of the high-level model. OOC meta-learning instead uses gradients of mapping functions of the low-level models as meta-data, which represent *how* the model learns, and retrains the gradients of the low-level models by the high-level model.

### Stress model selection

Finally, training should be stopped at a suitable point in order to avoid overfitting. This was achieved by stress model selection. Stress model selection is designed to basically recapitulate an OOD scenario by splitting the data into OOD train, OOD development, and OOD test sets as listed in Table 1; in this procedure, the data distribution for the development set differs from that of the training data, and the distribution of the test data set differs from both the training and development data. Section Algorithm provides further methodological details, covering data pre-processing, the core algorithm, model configuration, and implementation details.

**Table 1:** Data split scheme for stress model instance selection.

Data split	Common practice	Classic scheme applied in OOD	PortalCG	Specification
train	IID train	IID train	/	each batch includes data from the same gene family
	/	/	OOD train	data from different gene families are used among batches
dev	IID-dev	IID-dev	/	from the same gene family as that in the train set
	/	/	OOD-dev	from a different gene family from the training set
test	IID-test	/	/	from the same gene family as that in the training set
	/	OOD-test	OOD-test	from a different gene family from both OOD-dev and training set

### There are significantly unexplored dark gene families for small molecule binding

**Fig 2:**
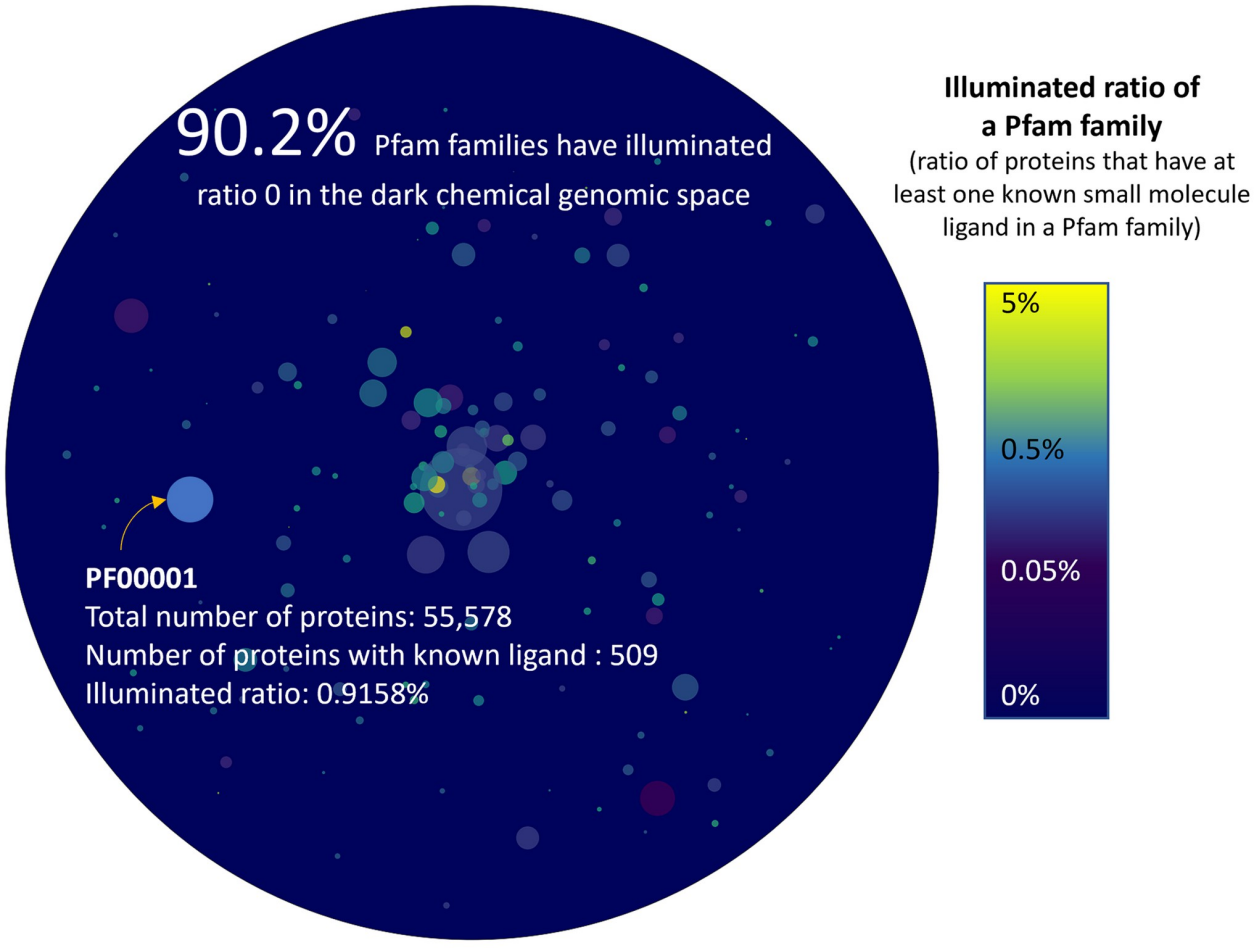
Dark protein space in terms of statistics. The fraction of proteins that have at least one known ligand in each Pfam family is graphically represented here. Each color bubble indicates a Pfam family, and the size of the bubble is proportional to the total number of proteins in that family. 1, 734 Pfam families have at least one known small molecule ligand. One can see that most Pfam families have less than 1% proteins with known ligands. Furthermore, around 90.2% of the total 17, 772 Pfam families remain completely dark, without any known ligand-binding information. These “dark regions” represent a vast untapped resource in drug discovery.

We inspected the known CPIs between (i) molecules in the manually-curated ChEMBL database, which consists of only a small portion of the possible chemical space, and (ii) proteins annotated in Pfam-A [30], which represents only a narrow slice of the whole protein sequence space. The ChEMBL26 [31] database supplies 1, 950, 765 chemicals paired to 13, 377 protein targets, constituting 15, 996, 368 known interaction pairs. Even for just this small portion of chemical genomics space, the fraction of unexplored gene families is enormous, as can be seen in the dark region in Fig 2. Approximately 90% of Pfam-A families do not have any known small-molecule binder. Even in Pfam families with annotated CPIs (e.g., GPCRs), there exists a significant number of “orphan” receptors with unknown cognate ligands (Fig 2). Because protein sequences in the dark gene families could be significantly different (beyond the point of homology) from those for the known CPIs, predicting CPIs for dark proteins is an archetypal, unaddressed OOD problem.

### PortalCG significantly outperforms state-of-the-art approaches to predicting CPIs of dark gene families

**Fig 3:**
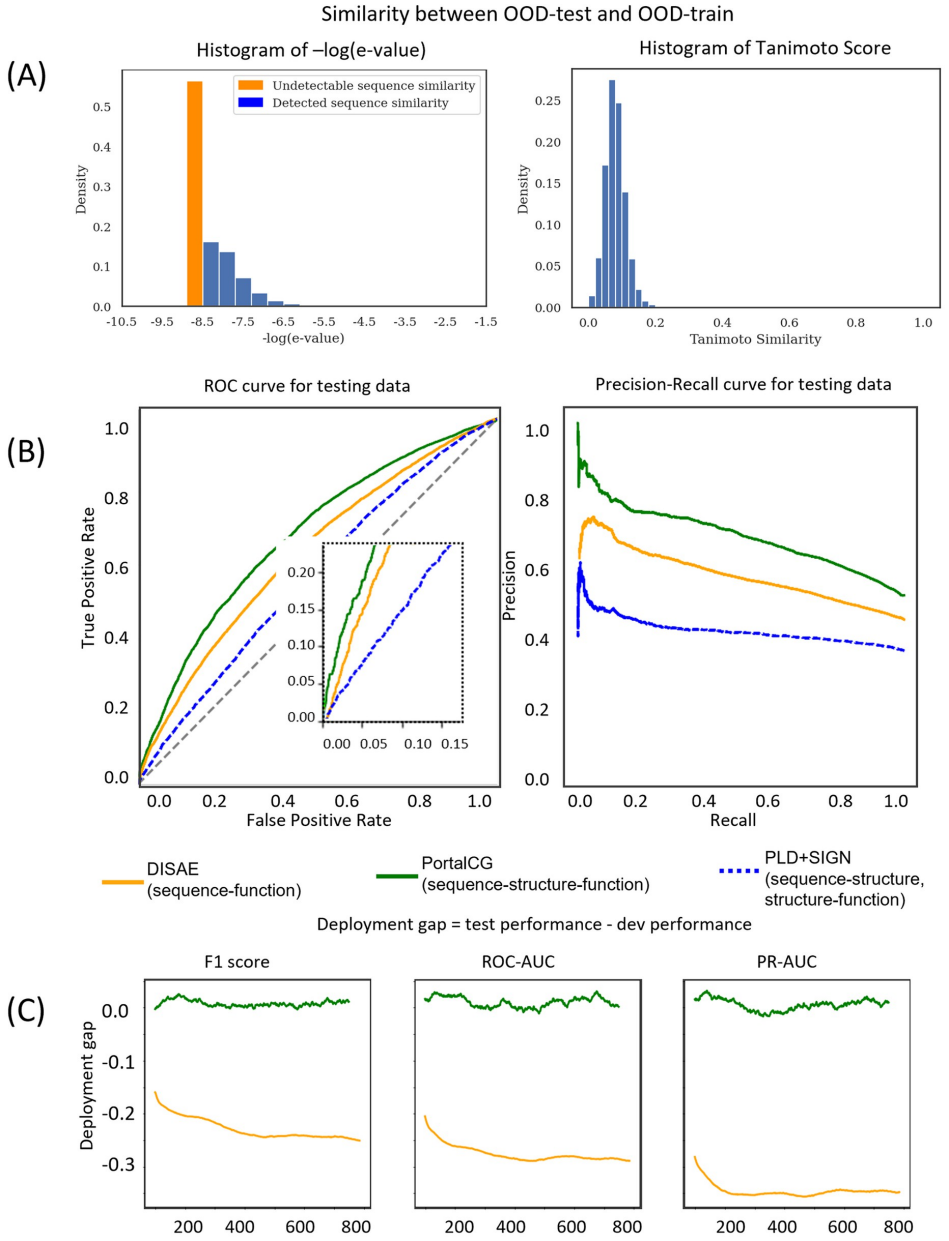
Performance comparison of PortalCG with the state-of-the-art methods DISAE and PLD+SIGN as baselines, using an OOD test with proteins in the test dataset coming from different Pfam families versus proteins in the training and validation datasets. (A) Histograms of protein sequence and chemical structure similarities between OOD-train and OOD-test. The majority of protein sequences in the training set do not have detectable similarity to proteins in the testing set. (B) Receiver Operating Characteristic (ROC) and Precision-Recall (PR) curves for the “best” model instance selected by the stress test. Due to the class-imbalanced active/inactive data, the PR curve is a more reliable measure than the ROC curve. (C) Deployment gaps of PoralCG and DISAE. The deployment gap of PortalCG is steadily around zero as the number of training steps increases, while the deployment performance of DISAE deteriorates.

Over the years, two major types of methodological approaches have developed for CPI predictions: those based on machine learning and on protein-ligand docking (PLD). A recent approach known as DISAE (distilled sequence alignment embedding) has been shown to outperform other leading deep learning methods for predicting CPIs of orphan receptors and is interpretable [1]. Because the neural network architecture of PortalCG is similar to that of DISAE, we used DISAE as the baseline against which to evaluate the performance improvement of PortalCG over the state-of-the-art machine learning method. PortalCG demonstrates superior performance in terms of both Receiver Operating Characteristic (ROC) and Precision-Recall (PR) curves when compared with DISAE, as shown in Fig 3(A). When the ratio of positive and negative cases is imbalanced, the PR curve is more informative than the ROC curve. The PR-AUC of PortalCG and DISAE is 0.714 and 0.603, respectively. In this regard, the performance gain of PortalCG (18.4%) is significant (p-value < 1e−40). Performance breakdowns for binding and non-binding classes can be found in Supplemental Fig A in S1 Text. PortalCG exhibits much higher recall and precision scores for positive cases (i.e., a chemical-protein pair that is predicted to bind) versus negative, as shown in Supplemental Fig A in S1 Text; this is a highly encouraging result, given that there are many more negative (non-binding) than positive cases in reality. The deployment gap, shown in Fig 3(B), is steadily around zero for PortalCG; this promising finding means that we can expect that, when applied to the dark proteins, the performance will be similar to that measured using the development data set.

With the advent of high-accuracy protein structural models, predicted by AlphaFold2 [5], it now becomes possible to use reversed protein-ligand docking (PLD) [20] to predict ligand-binding sites and poses on dark proteins on a genome-wide scale. In order to compare our method with the reversed protein-ligand docking approach, blind PLD to proteins in the benchmark was performed via AutoDock Vina [32] followed by protein-ligand binding affinity prediction using a leading graph neural network-based method called SIGN [33]; we denote this approach “PLD+SIGN”. The binding affinities predicted by SIGN were more accurate than the original scores from AutoDock Vina (Supplemental Fig B in S1 Text). The performance of PLD+SIGN was compared with that of PortalGC and DISAE. As shown in Fig 3(A), both ROC and PR for PLD+SIGN are significantly worse than for PortalGC and DISAE. PortalCG’s end-to-end sequence-structure-function learning could be a more effective strategy in terms of both accuracy and efficacy, especially for remaining half of dark human proteins that cannot be reliably predicted by AlphaFold2: protein structure information is not used as a fixed input, but rather as an intermediate layer that can be tuned using various structural and functional information. Furthermore, the inference time of PortalCG for predicting a CPI is several orders of magnitude faster than that needed for PLD calculations. For example, it takes approximately 1 millisecond for PortalCG to predict a ligand binding to DRD2, while AutoDock Vina needs around 10 seconds to dock a ligand to DRD2, excluding the time for defining the binding pocket.

### Both the STL and OOC-ML stages contribute to the improved performance of PortalCG

To gauge the potential contribution of each component of PortalCG to the overall system effectiveness in predicting CPIs for dark proteins, we undertook an ablation study wherein we systematically compared the four models shown in Table 2. Details of the exact model configurations for these experiments can be found in the Supplemental Table A in S1 Text. As shown in Table 2, Variant 1, with a higher PR-AUC compared to the DISAE baseline, is the direct gain from transfer learning through 3D binding site information, all else being equal; yet, with transfer learning alone and without OOC-ML as an optimization algorithm in the protein function CPI prediction (i.e., Variant 2 versus Variant 1), the PR-AUC gain is minor. Variant 2 yields a 15% improvement while Variant 1 achieves only a 4% improvement over DISAE. PortalCG, in comparison, has the best PR-AUC score. With all other factors held constant, the advantage of PortalCG appears to be the synergistic effect of both 3D binding site encoding and OOC-ML. The performance gain measured by PR-AUC under a shifted evaluation setting is significant (p-value < 1e−40), as shown in Supplemental Fig C in S1 Text. We find that stress model selection is able to mitigate potential overfitting problems, as expected. Training curves for the stress model selection are in Supplemental Fig D in S1 Text. As shown in Supplemental Fig D in S1 Text, the baseline DISAE approach tends to over-fit with training, and IID-dev performances are all higher than PortalCG but deteriorate in OOD-test performance. Hence, the deployment gap for the baseline is -0.275 and -0.345 on ROC-AUC and PR-AUC, respectively, while the respective PortalCG deployment gaps are approximately 0.01 and 0.005.

**Table 2:** Ablation study of the performance of PortalCG.

Models	Configuration	OOD-test set		Deployment gap	
		ROC-AUC	PR-AUC	ROC-AUC	PR-AUC
PortalCG	PortalCG with all components	0.677±0.010	0.714±0.010	0.010±0.009	0.005±0.010
DISAE	PortalCG w/o STL or OOC-ML	0.636±0.004	0.603±0.005	-0.275±0.016	-0.345±0.012
Variant 1	PortalCG w/o OOC-ML	0.661±0.004	0.629±0.005	/	/
Variant 2	PortalCG w/o STL	0.654±0.062	0.698±0.015	/	/
PLD+SIGN	/	0.569	0.433	/	/

### PortalCG is competitive in virtual screening for novel compounds

Given that the pretraining, OOC-ML, and stress tests were only applied to proteins, the current PortalCG method primarily focuses on exploring the dark protein space instead of new chemical space. Nevertheless, we examined whether PortalCG could improve the performance of compound screening for novel chemicals. We employed a widely used DUD-E benchmark that included eight protein targets along with their active compounds and decoys [34], and we compared the performance of PortalCG with that of PLD. We used DUD-E chemicals as a testing set. We trained PortalCG by excluding target proteins in the training/validation sets, with all chemicals in the training/validation set being dissimilar to those in the testing set (Tanimoto Coefficient (TC) less than 0.3 or 0.5). Under these chemical similarity thresholds, the false positive rate in the training/validation set was higher than 95.0%, assuming a ratio of actives to inactives of 1:50 (Supplemental Fig E in S1 Text).

As shown in Table 3, except for the targets kif11 and gcr, PortalCG could surprisingly outperform AutoDock Vina on the other remaining six targets, in terms of enrichment factors (EFs). Similarly, PortalCG exhibited higher EFs than PLD-SIGN on six proteins. For an EF of 1%, the compound screening performance of PortalCG on 87.5% and 100.0% of targets is better than random guesses (EF = 1.0) when the chemical similarity between the queries and the training data is 0.3 and 0.5, respectively. In contrast, only 50.0% and 75.0% of targets are better than a random guess for AutoDock Vina and PLD+SIGN, respectively. These results imply that PortalCG has learned certain patterns of CPIs, even though the chemical OOD issues were not explicitly modeled. Different from PLD, whose EFs varied greatly across targets, the variance of EFs was relatively small for PortalCG across the targets, suggesting that the model is not biased towards certain types of proteins (akt1 is a kinase, cxcr4 is a chemokine receptor, and gcr is a nuclear receptor, etc.). Thus, PortalCG is complementary with PLD, and has the potential to improve the capability of virtual compound screening—particularly for dark proteins whose reliable structures are not available.

**Table 3:** Compound screening performances evaluated using the DUD-E benchmark. For “PortalCG-0.3”, the similarities between chemicals in the training/validation set and those in the testing set are less than 0.3 of the Tanimoto Coefficient (TC). For ‘PortalCG-0.5’, the similarities between chemicals in the training/validation set and those in the testing set are less than 0.5 of the TC. The best performance is accentuated in **bold**.

	EF-1%				EF-20%			
	AutoDock Vina	PLD-SIGN	PortalCG-0.3	PortalCG-0.5	AutoDock Vina	PLD-SIGN	PortalCG-0.3	PortalCG-0.5
**akt1**	0.00	**14.42**	1.36	11.24	1.52	3.12	2.61	**3.88**
**ampc**	0.00	0.00	2.04	**4.08**	1.25	0.39	0.31	**2.14**
**cp3a4**	0.60	3.03	2.50	**10.00**	1.65	**2.07**	0.63	1.38
**cxcr4**	0.00	1.64	5.00	**10.00**	0.87	1.89	2.13	**2.25**
**gcr**	**10.43**	2.49	4.65	9.69	1.98	2.03	**2.50**	1.96
**hivpr**	4.10	5.02	0.75	**13.62**	2.31	2.34	1.87	**2.84**
**hivrt**	4.77	0.47	1.18	**8.28**	2.20	1.21	0.15	**2.59**
**kif11**	**23.15**	13.71	1.72	3.45	**3.66**	3.60	1.60	1.08

### PortalCG is able to screen selective, multi-targeted compounds that bind dark proteins and feature novel scaffolds

Opioid use disorder (OUD) is an overwhelming healthcare and economic burden. Although several pharmaceutical treatments for OUD exist, they are either restricted in usage or limited in effectiveness. Dopamine D1 and D3 receptors (DRD1 and DRD3) have been identified as potential drug targets for OUD. DRD1 partial agonists and antagonists alter the rewarding effects of drugs, while DRD3 antagonists reduce drug incentive and behavioral responses to drug cues [35, 36]. Moreover, recent evidence suggests that simultaneous targeting of DRD1 and DRD3 may be an effective OUD therapeutic strategy as the combination of a DRD1 partial agonist and a DRD3 antagonist reduced cue-induced relapse to heroin in rats [37]. By contrast, dopamine D2 receptor (DRD2) antagonism is associated with cataleptic side effects which limit the use of DRD2 antagonists as OUD therapeutics [38]. Thus, selective DRD1 and DRD3 dual-antagonists could be an effective strategy for OUD treatment [39]. Because there are multiple dopamine receptors (especially DRD2) that are similar to D1R and D3R, it is challenging to develop a selective dual-antagonist for DRD1 and DRD3. PortalCG may provide new opportunities for OUD polypharmacology.

**Fig 4:**
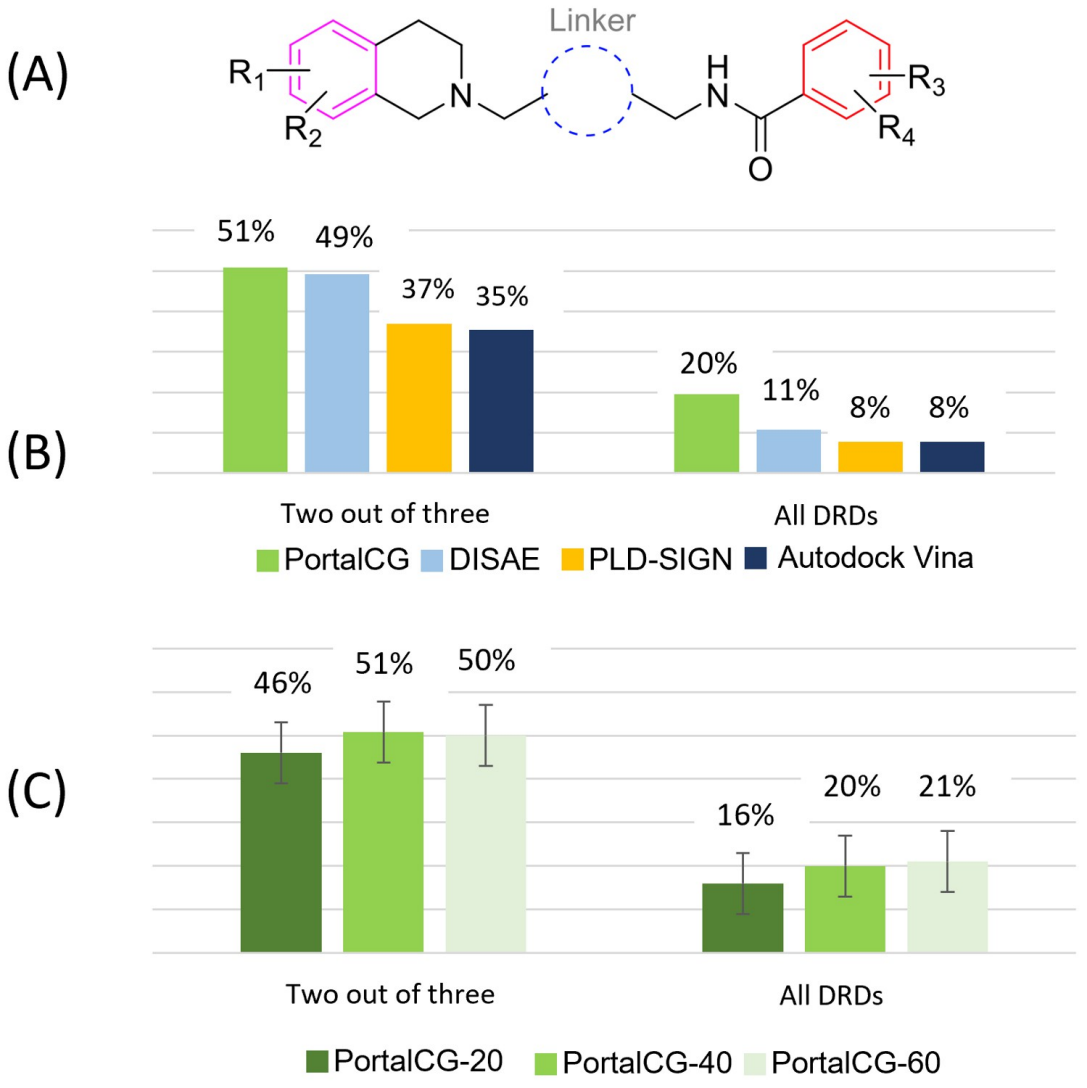
Performance comparison of PortalCG with the state-of-the-art methods for designing selective dual-DRD antagonists. (A) The chemical scaffold on which 65 compounds were synthesized as potential selective dual-DRD1/DRD3 antagonists. Tens of thousands of chemicals can be generated from the different combination of four functional groups R1, R2, R3, and R4 and a linker group. (B) The prediction accuracy of DRD binding profile classification. Note that a significant difference between PortalCG’s performance relative to the next-best method (DISAE) emerges in a task involving correct prediction of all three DRDs (right-hand side), versus just two of the three (DRD1, DRD2, and DRD3). (C) The performance of PortalCG when sequence similarities between the proteins in the training/validation set and DRD1/DRD2/DRD3 were less than 20%, 40%, and 60%, respectively. The performance was measured by the accuracy of a three-label classifier. “Two out of three” and “all DRDs” represented the accuracy when two labels and all three labels were predicted correctly.

We synthesized 65 compounds based on the scaffold shown in Fig 4A, which combines structural features of the DRD1 antagonist (-)-stepholidine with a DRD3 antagonist pharmacophore, and we then determined their binding affinities to DRD1, DRD2, and DRD3, respectively (Supplemental Table B in S1 Text). Tens of thousands of possible chemical structures could be derived from different combinations of R1, R2, R3, R4, and linker functional groups, as marked in Fig 4A. We have little *a priori* knowledge of what is an optimal combination of functional groups for a dual-DRD1/DRD3 antagonist. If we define an acceptable dual-DRD1/DRD3 antagonist as a compound whose binding affinities are less than 100 nM of *K*_*i*_ to both DRD1 and DRD3, but higher than 100 nM of the *K*_*i*_ to DRD2, then only 10 compounds were found to satisfy this condition (successful rate of 15.4%) among the 65 synthesized compounds. For the DRD1 antagonists with the *K*_*i*_ lower than 100 nM, only 46.4% of them had *K*_*i*_ lower than 100 nM for DRD3. These observations suggested that our current knowledge is limited for effectively designing selective dual-DRD1/3 antagonists using existing scaffolds, let alone under a novel scaffold. The question is if we can use computational methods, especially PortalCG, to identify selective dual-DRD1/3 antagonists with a novel scaffold. We performed a rigorous blind test to validate the performance of PortalCG for this purpose. In the evaluation of PortalCG and DISAE, all of the chemicals in the training data had different scaffolds from 65 test compounds, i.e., an OOD scenario on the chemical side [40]. Three models were trained with the sequence similarity between DRD1/2/3 and proteins in the training/validation data ranging from 20% to 60%. The performance was measured by the accuracy of a three-label classifier. When the sequence identifies between DRD1/2/3 and the proteins in the training/validation set were less than 40%, PortalCG achieved 20.0% and 50.7% success rates for the cases where all DRDs and any two of them were predicted correctly using aforementioned criteria, respectively, for the top 30 ranked compounds (Fig 4B). The success rate of PortalCG that was trained with OOD data was higher than that based on the random selection out of 65 compounds. Decreasing the sequence identifies between the proteins in the training/validation set and DRD1/2/3 from 40% to 20% only slightly lower the accuracy of PortalCG, as shown in Fig 4C. The performance drops were not statistically significant (p-value > 0.05). Increasing the sequence identities from 40% to 60% also did not significantly change the accuracy. Thus, PortalCG by design was robust to OOD data.

We compared PortalCG with three baselines—DISAE, PLD+SIGN, and AutoDock Vina [32]. The crystal structures of DRD1 (PDB id: 7JOZ), DRD2 (PDB id: 6CM4), and DRD3 (PDB id: 3PBL), which were co-crystallized with ligands, were used in the docking calculations. The 65 compounds were docked to the pre-defined binding pocket based on the co-crystallized ligand. The order of accuracy follows PortalCG > DISAE > PLD-SIGN > AutoDock Vina, as shown in Fig 4B. This observation is consistent with our benchmark studies. Note that the protein-ligand complex structure was used only for the baseline PLD models and this information was not used for PortalCG and DISAE.

### Illuminating the undruggable human genome for drug repurposing

To further gauge the potential applications of PortalCG, we explored potential drug lead compounds for undrugged disease genes in the dark human genome, and prioritized undrugged genes that can be efficaciously targeted by existing drugs. It is well known that only a small subset of the human genome is considered druggable [41]. Many proteins are deemed “undruggable” because there is no information on their ligand-binding properties or other interactions with small-molecule compounds (be they endogenous or exogenous ligands). Here, we built an undruggable human disease protein database by removing the druggable proteins in Pharos [42] and Casas’s druggable proteins [43] from human disease associated genes [17]. A total of 12,475 proteins were included in our disease-associated undruggable human protein list. We applied PortalCG to predict probabilities for these putatively undruggable proteins to actually be able to bind to drug-like molecules. Around 6,000 drugs from the Drug Repurposing Hub [44] were used in this screening. The proteins that could bind to a small-molecule drug were ranked according to their prediction scores, and 267 of them have a false positive rate lower than 2.18e-05, as listed in Supplemental Table C in S1 Text. Table 4 shows the statistically significantly enriched functions of these top-ranked proteins, using the Database for Annotation, Visualization and Integrated Discovery (DAVID) utility [45]. The most enriched proteins are involved in alternative splicing of mRNA transcripts. Malfunctions in alternative splicing are linked to many diseases, including several cancers [46, 47], Alzheimer’s disease [48], and insulin resistance and type-2 diabetes [49]. However, pharmaceutical intervention and modulation of alternative splicing is a challenging task, given the intricacy of these pathways. Identifying new drug targets and their lead compounds for targeting alternative splicing pathways may open new doors to developing novel therapeutics for complex diseases with few treatment options. In addition, we identified several transcription factors and proteins otherwise related to cellular transcription activities; these are listed in Supplemental Table D in S1 Text, along with their predicted ligands.

**Table 4:** Functional annotation enrichment for undruggable human disease associated proteins selected by PortalCG.

DAVID Functional annotation enrichment analysis				
Enriched terms in UniProtKB keywords	Number of proteins involved	Percentage of proteins involved	P-value	Modified Benjamini p-value
Alternative splicing	171	66.5	7.70E-07	2.00E-04
Phosphoprotein	140	54.5	2.60E-06	3.40E-04
Cytoplasm	91	35.4	1.30E-05	1.10E-03
Nucleus	93	36.2	1.20E-04	8.10E-03
Metal-binding	68	26.5	4.20E-04	2.20E-02
Zinc	48	18.7	6.60E-04	2.90E-02

**Fig 5:**
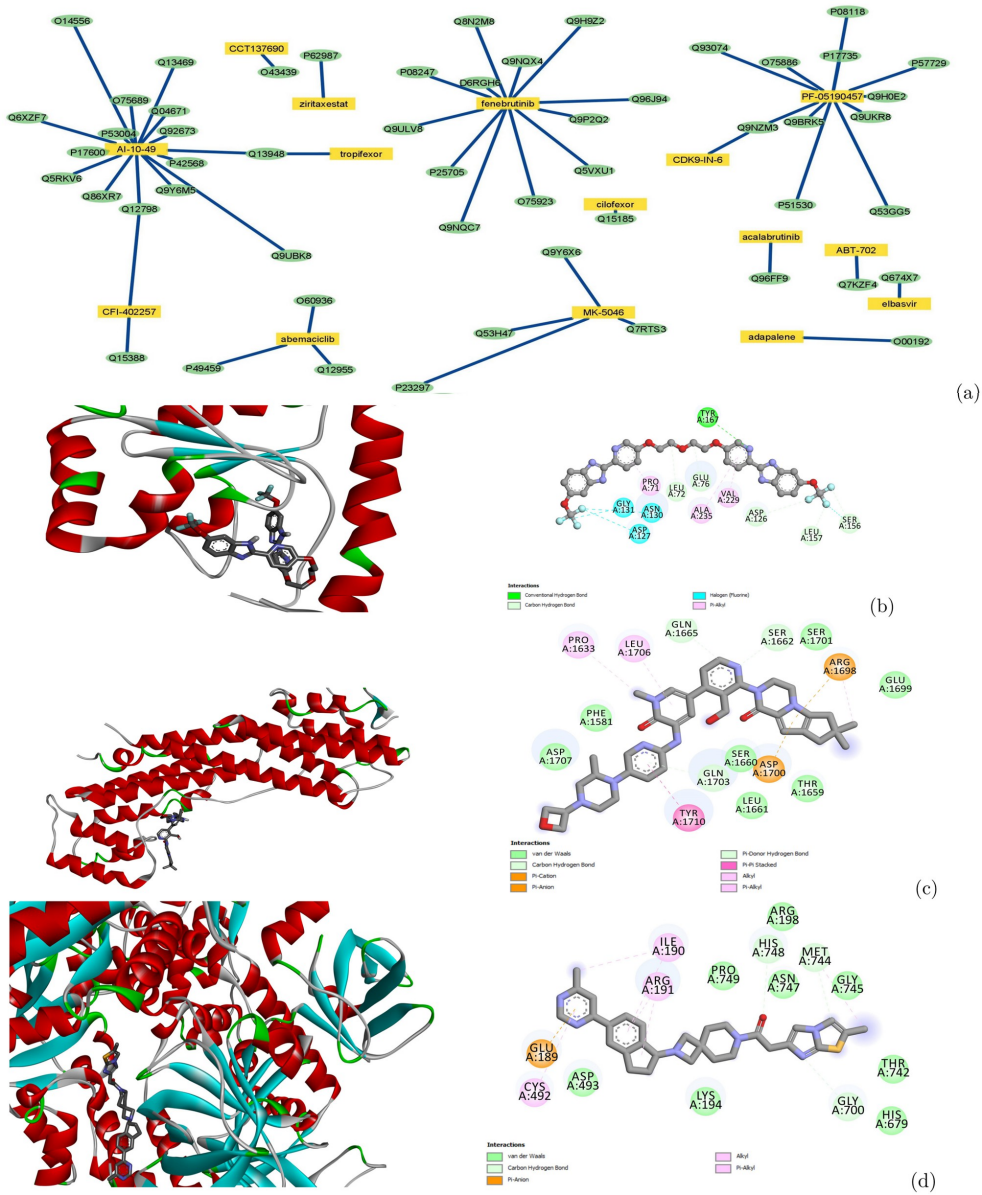
Drug-target interaction network for proteins associated with Alzheimer’s disease and docking poses for representative drug-target pairs calculated by Autodock Vina. (a) Drug-target interaction network predicted by PortalCG. Yellow rectangles and green ovals represent drugs and targets, respectively. (b) Docking pose and ligand binding interactions between protein TIR domain-containing adapter molecule 2 (Uniprot: Q86XR7) and AI-10–49. (c) Docking pose and ligand binding interactions between protein Unconventional myosin-Vc (Uniprot: Q9NQX4) and fenebrutinib. (d) Docking pose and ligand binding interactions between DNA replication ATP-dependent helicase/nuclease (Uniprot: P51530) and PF-05190457.

Diseases associated with these 267 human proteins are also listed in Table 5. Since one protein is always related to multiple diseases, these diseases are ranked by the number of their associated proteins. The most highly-ranked diseases tend to be related to cancer development. We find that 21 drugs that are approved or in clinical development are predicted to interact with these proteins (Supplemental Table E in S1 Text). Several of these drug compounds are highly promiscuous. For example, AI-10–49, a molecule that disrupts protein-protein interaction between CBF*β*-SMMHC and the tumor suppressor RUNX1 [50], may bind to more than 60 other proteins. The off-target binding profile of these proteins may provide invaluable information on potential side-effects and opportunities for drug repurposing and polypharmacology. A drug-target interaction network, built for predicted positive proteins associated with Alzheimer’s disease, is shown in Fig 5. The target proteins in this network were selected based on a threshold of 0.67. The length of the edges in this network was decided by the prediction scores for these drug-target pairs. The longer the edge is, the lower confidence of the prediction is. Thus if a higher threshold was applied, fewer drug-target pairs will appear in this network. In order to validate the binding activity between the drugs and targets in this network, PLD was performed between the three most promiscuous drugs—AI-10–49, fenebrutinib, and PF-05190457—and their predicted targets. Only those targets with known PDB structures or reliable AlphaFold structural models were used in the docking. Docking scores for the 21 drug-target pairs are listed in Supplemental Table F in S1 Text. For each of the three drugs, the target with the lowest docking score (the highest binding affinity) was selected as a representative. Docking conformations and interactions between the drugs and their representative targets are shown in Fig 5. Functional enrichment, disease associations, and top-ranked drugs for the undruggable proteins with well-studied biology (classified as Tbio in Pharos), as well as those excluding Tbio, are given in Supplemental Tables G-K in S1 Text.

**Table 5:** These highly-ranked diseases are associated with undruggable human disease proteins, as selected by PortalCG.

DiseaseName	# of undruggable proteins associated with disease
Breast Carcinoma	90
Tumor Cell Invasion	86
Carcinogenesis	83
Neoplasm Metastasis	75
Colorectal Carcinoma	73
Liver Carcinoma	66
Malignant Neoplasm of Lung	56
Non-Small Cell Lung Carcinoma	56
Carcinoma of Lung	54
Alzheimer’s Disease	54

## Conclusion

This work has confronted the challenge of exploring dark proteins by recognizing it, fundamentally, as an OOD generalization problem in machine learning, and by developing a new deep learning framework to treat this type of problem. Though the applications given in this paper are all biological systems, we propose that PortalCG is a general framework that enables systematic control of the generalization risk inherent to OOD model training and prediction. Systematic examination of the PortalCG method revealed its superior performance compared to (i) a state-of-the-art deep learning model (DISAE), and (ii) an AlphaFold2-enabled, GNN-scored, structure-based reverse docking approach, using classical protein-ligand docking methods. Compared to those methods, PortalCG showed significant improvements in terms of both sensitivity and specificity, as well as close to zero deployment performance gap. The neural network architecture of PortalCG is similar to DISAE, and its performance improvement (over DISAE) mainly stems from 3D binding site-enhanced pre-training (step-wise transfer learning) and OOC-ML optimization. Both PortalCG and DISAE outperform PLD-based methods by obviating the inherent limitations of PLD. Applications of PortalCG to OUD polypharmacology and drug repurposing targeting of hitherto undruggable human proteins afford novel directions in drug discovery. For example, there are numerous predictions for potential drug leads (and pathways to target for intervention) that can now be experimentally tested and pursued, based on the predicted dark protein targets of the top-three ligands that we identified above via PortalCG.

PortalCG can be further improved along several directions. In terms of protein sequence modeling, additional *a prior* knowledge of protein structure and function can be incorporated into the pre-training or supervised multi-task learning. Also, the current architecture of PortalCG mainly focuses on addressing the OOD problem from the perspective of protein space but not chemical space. New methods for modeling chemical structures alone, or the joint space of chemicals and proteins, will no doubt improve CPI predictions for hitherto unseen, novel chemicals. Future directions can include novel representation schemes for 3D chemical structures [51] at the sub-molecular level of scaffold and chemical moieties, pre-training of the chemical space [52], and few-shot learning [53], as well as explicitly modeling inter-atomic interactions between target amino acid residues and chemical/drug moieties. Finally, also note that the existing PortalCG framework treats CPI prediction as a binary classification problem, but this can be better reformulated as a regression model for predicting binding affinities. By defining domain-specific pre-training and down-stream supervised learning tasks, PortalCG can be envisaged as a general framework to explore the functions of understudied proteins, including their universe of protein-protein interactions and protein-nucleic acid recognition.

## Methods

**Fig 6:**
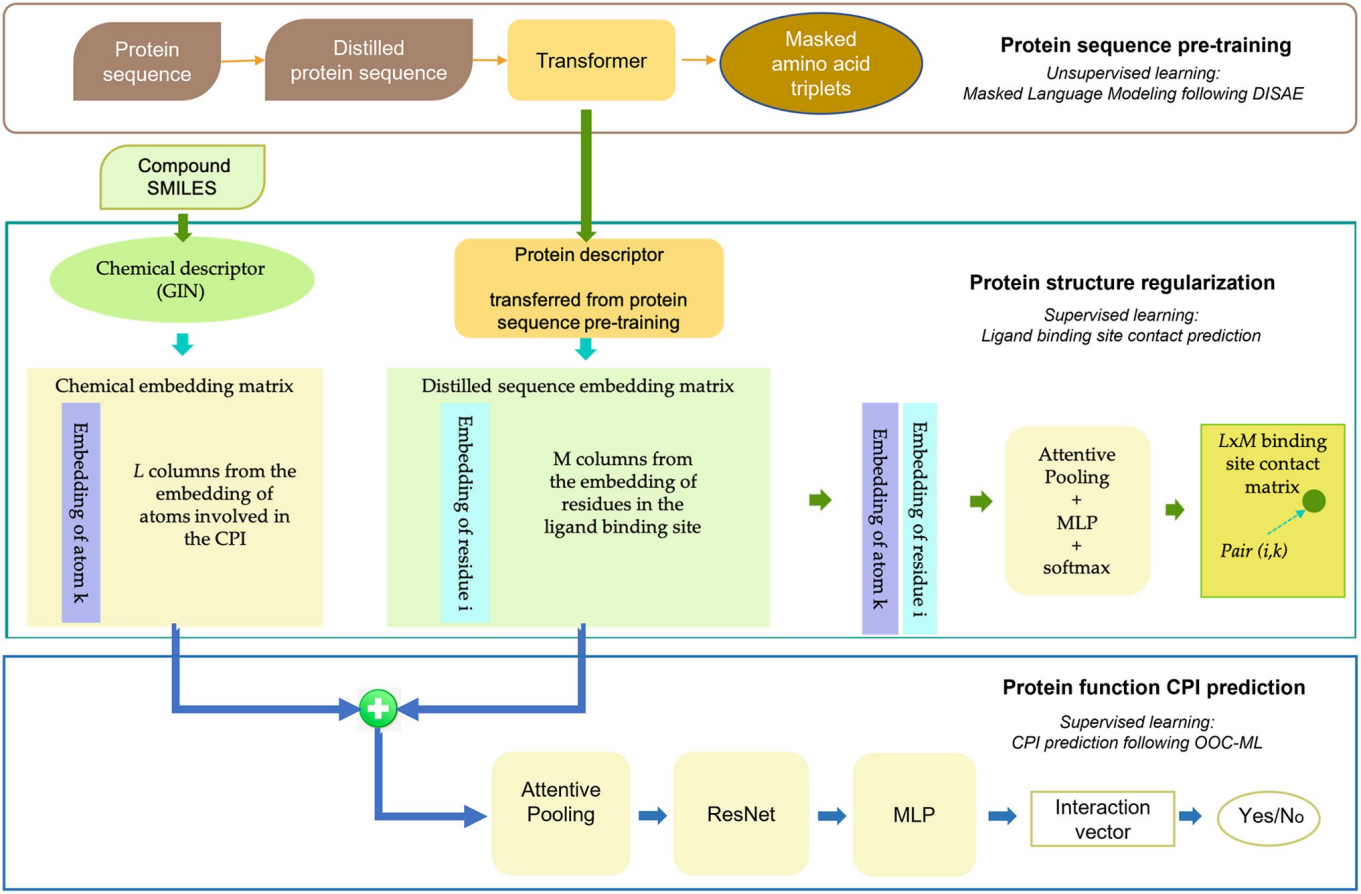
Illustration of PortalCG architecture in terms of its three stages of training. The architecture of protein sequence pre-training used transformer-based and masked language modeling as detailed in [1]. The pre-trained protein descriptor was then used in binding site enhanced sequence pre-training. In this stage, the task was to predict amino acid residue and ligand atom distance matrices. Finally, protein descriptors that were pre-trained and regularized in the previous two stages were concatenated with chemical descriptors via an attention network to predict CPIs. Chemical structures were represented by GIN [54], a graph neural network model (see text). The second and third stages had the same model architecture but the model parameters were transferred from the second to the third stages. OOC-ML as an optimization algorithm was not a model architecture component, and only used in the CPI prediction.

PortalCG, as a system-level framework, involves collaborative new design from data preprocessing, data splitting to model initialization, and model optimization and evaluation. The overall pipeline of the framework is schematized in Fig 1. The model architecture adopted in PortalCG mostly follows DISAE, as shown in Fig 6.

### Datasets

PortalCG was trained using four major databases, namely Pfam [30], the Protein Data Bank (PDB) [55], BioLp [56] and ChEMBL [31]. The data were pre-processed as follows.

- Protein sequence data. All sequences from Pfam-A families are used to pretrain the protein descriptor following the same setting as in DISAE [1], which distills the original sequence into an ordered list of amino acid triplets by extracting evolutionarily important positions from a multiple sequence alignment
- Protein structures. Our protein structure dataset contains 30,593 protein 3D structures, 13,104 ligands, and 91,780 ligand-binding sites. Binding sites were selected according to the annotation from BioLip (updated to the end of 2020). Binding sites which contact either DNA/RNA or metal ions were not included. If a protein has more than one ligand, multiple binding pockets were defined for this protein. For each binding pocket, pairwise distances between the *C*_*α*_ atoms of amino acid residues of the binding pocket were calculated. In order to obtain the distances between the ligand and its surrounding binding site residues, the distances between atom *i* of the ligand and each atom *j* in the binding-pocket residue were calculated and the smallest such distance was selected as “the” distance between atom *i* and residue *j*. In order to obtain the sequence feature of the binding site residues, in the proper DISAE protein sequence representation [1], binding site residues obtained from PDB structures (queries) were mapped onto the multiple sequence alignments of its corresponding Pfam family. First, a profile HMM database was built for the whole Pfam family. The tool hmmscan [57] was applied to search the query sequence against this profile database to decide which Pfam family it belongs to. For those proteins with multiple domains, more than one Pfam families were identified. Then the query sequence was aligned to the most similar sequence in the corresponding Pfam family by using phmmer. Aligned residues on the query sequence were mapped to the multiple sequence alignments of this family according to the alignment between the query sequence and the most similar sequence
- Chemical genomics data. CPI classification prediction data is the whole ChEMBL26 [31] database, where the same threshold for defining positive and negative labels was used as that in creating DISAE [1]. Log-transformation was performed for activities reported in *pK*_*d*_, *pK*_*i*_ or *pIC*_50_. The activities on a log-scale were then binarized, with protein-ligand pairs considered “active” if *pIC*_50_ > 5.3, *pK*_*d*_ > 7.3 or *pK*_*i*_ > 7.3 [1]

All of the data described above were split into training, validation, and testing sets. Data-split statistics are shown in Table 6, and other data statistics are provided in Fig 2.

**Table 6:** Data statistics for each training stage.

dataset	usage in PortalCG	count sample size			note
Pfam 33.1	STL, the first pretraining step to train DISAE	# Pfam families		17,772	random split in training and testing
		# sequences		54,409,760	
PDB	STL, the second pretraining step to learn contact map between amino acid residues and ligand atoms at binding sites	train	# Pfam families	319	Pfam families in OOD-dev and OOD-test are held out from PDB pre-training.
			# proteins	5,926	
			# binding sites (protein-ligand pairs)	6,896	
			# chemical	3,168	
		test	# Pfam families	733	
			# proteins	1,497	
			# binding sites (protein-ligand pairs)	1,573	
			# chemical	670	
ChEMBL 26	OOC-ML	ODD-train	# protein-ligand pairs	1,672,277	within each split (OOD-train/IID-dev/OOD-dev/OOD-test), the data is random split into support and query sets in a ratio of 5:1 for each Pfam family unless there are only one class (binding or not) of data
			# chemical	478,939	
			# Pfam families	333	
		IID-dev	# protein-ligand pairs	6,536	
			# chemical	6,096	
			# Pfam families	333	
			# Pfam families overlapping with OOD-train	333	
		OOD-dev	# protein-ligand pairs	165,655	
			# chemical	98,975	
			# Pfam families	701	
			# Pfam families overlapping with OOD-train	0	
		OOD-test	# protein-ligand pairs	162,354	
			# chemical	104,299	
			# Pfam families	700	
			# Pfam families overlapping with OOD-train	0	
			# Pfam families overlapping with OOD-dev	0	

65 compounds were synthesized for testing DRD1/2/3 binding activities. The procedures for the compound synthesis were detailed in Supplemental S1 Text Section 1.6, Scheme 1–5. DRD binding assays and Ki determinations were performed by the Psychoactive Drug Screening Program (PDSP).

For illuminating undruggable human proteins, around 6,000 drugs are collected from CLUE [44]. 12,475 undruggable proteins are collected by removing the druggable proteins in Pharos [42] and Casas’s [43] druggable proteins sets from human disease associated genes [17].

### Algorithm

#### Chemical representation

We represent a chemical compound as a graph, and its embedding is learned using Graph Isomorphism Network (GIN) [54], which is designed to maximize the representational (or discriminative) power of a Graph Neural Network (GNN) based on the Weisfeiler-Lehman (WL) graph isomorphism test. GIN is a common choice as a chemical descriptor [40].

#### Protein sequence pre-training

PortalCG’s protein descriptor is pretrained from scratch, following exactly the approach of DISAE [1] on whole Pfam families, making it a universal protein language model. DISAE, which was inspired by recent success in self-supervised learning of unlabeled data in Nature Language Processing (NLP), features a novel method, termed DIstilled Sequence Alignment Embedding (DISAE), for protein sequence representation. DISAE can utilize all protein sequences to capture functional information without any knowledge of their structure and function. By incorporating biological knowledge into the sequence representation, DISAE can learn functionally important information about protein families that span a wide range of protein sequence space. In contrast to existing sequence pre-training strategies, which use original protein sequences as input [27], DISAE distills the original sequence into an ordered list of triplets by extracting evolutionary important positions from a multiple sequence alignment (including insertions and deletions). Next, long-range residue-residue interactions can be learned via the Transformer module in ALBERT ([10]; itself derived from the highly successful Bidirectional Encoder Representations from Transformers [BERT] language model). A self-supervised masked language modeling (MLM) approach was used at this stage. In the MLM, 15% triplets are randomly masked and assumed that they are unknown; then, the remaining triplets are used to predict what the masked triplets are.

#### Protein structure regularization

With the protein descriptor pretrained using the sequences from the whole of Pfam, chemical descriptors and a distance learner were plugged in to fine-tune the protein representation. Specifically, the distance learner follows AlphaFold [4], which formulates a multi-way classification on a distogram. Based on the histogram of distances between amino acids and ligand atoms, a histogram equalization (https://en.wikipedia.org/wiki/Histogram_equalization) method was applied to formulate a 10-way classification on our binding site structure data, as in Supplemental Fig F in S1 Text. Since protein and chemical descriptors output position-specific embeddings of a distilled protein sequence, and all of the atoms of a chemical compound, we used simple vector operations to create pair-wise interaction feature descriptions of the binding sites. Specifically, a matrix multiplication was used to select embedding vectors of each binding-site residue and atom (this step can be thought of as applying a filter); then, multiplication and “broadcasting” the selected embedding vectors into a symmetric tensor was performed as shown in the following, where *H* is an embedding matrix of size (*number*_*of*_*residues*, *embedding*_*dimension*) [for the target binding-site residues] or (*number*_*of*_*atoms*, *embedding*_*d*_*imension*) [for the ligand compound], and *A* is the selector matrix [58],

$$H_{binding\_site}^{protein}=A^{protein}*H_{full\_distilled}^{protein}$$

$$H_{binding\_site}^{chemical}=A^{chemical}*H_{full\_chemical\_graph}^{chemical}$$

$$H_{binding\_site}^{interaction}={\left(H_{binding\_site}^{protein}\right)}^{T}*H_{binding\_site}^{chemical}$$

The final pair-wise interaction feature tensor, $H_{binding\_site}^{interaction}$, was fed into an Attentive Pooling [59] layer followed by a generic feed-forward layer for the final 10-way classification. Further details about the model architecture and configuration can be found in Supplemental Table A in S1 Text and Fig 6. The intuition for using a relatively simple form of the distance learner is to place all the “stress” of learning on the shared protein and chemical descriptors, which at any rate will carry information across the end-to-end neural network. Again, with standard Adam optimization, shifted evaluation was used to select the “best” instance. Two versions of distance structure prediction were implemented, one formulated as a binary classification (i.e. contact prediction), and the other formulated as a multi-way classification (i.e. distogram prediction).

#### Out-of-cluster Meta Learning (OOC-ML)

With a fine-tuned protein descriptor for the protein function space, a binary classifier is then utilized; this step takes the form of a ResNet [60] built with two linear layers, as shown in Supplemental Table A in S1 Text and Fig 6. What plays a major role in this phase is the optimization algorithm OOC-ML, shown in pseudocode **Algorithm 1** and Fig 1. The first level (low level) model training is captured in lines 4–9, and line 10 shows ensemble training of the second level (higher-level) models. Note that other variants could be derived by changing the sampling rules (line 3 and 5) and/or the second-level ensemble update rule (line 10).

**Algorithm 1**: Out-of-cluster (OOC) Meta-learning in PortalCG

**input**: *p*(*D*): CPI data distribution over the whole of Pfam, where each

    *D*_*i*_ ∈ **D** is a set of CPI pairs for a given family, *pfam*_*i*_;

    *α*, *β*: learning step-size hyperparameters;

    *L*: number of optimization steps in each round of first-level training;

    *T*: number of the second-level training steps;

    *K*: number of points sampled from a local neighborhood

**output**: *θ*: set of trained weights for the whole model

1 Initialize whole-model weights, *θ* (with weights transferred from portal for protein and chemical descriptors, and randomly initialized weights for binary classifier)

2 **for** *t in T* **do**

3  Sample a *D*_*i*_ ∼ *p*(*D*);

4  **for** *l in L* **do**

5   Sample a positive-negative balanced mini-batch of *K* pairs in *D*_*i*_;

6   **for** *point*_*j*_ *in* *D*_*i*_ **do**

7    Evaluate $\nabla_{\theta }L_{point_{j}}\left(f_{\theta }\right)$ with respect to *K* examples;

8    Compute adapted parameters with gradient descent: $\theta_{i}^{{}^{\prime }}=\theta -\alpha \nabla_{\theta }L_{point_{j}}\left(f_{\theta }\right)$;

9   **end**

10   Update $\theta \leftarrow \theta -\beta \nabla_{\theta }\sum_{D_{i}∼p\left(D\right)}L_{point_{j}}\left(f_{\theta }^{{}^{\prime }}\right)$;

11  **end**

12 **end**

#### Stress model instance selection

In classic training schemes, a common practice is that there are 3-split data sets, namely “train set”, “development (dev) set” and “test set”. The training set, as the name suggests, is used to train an ML model. The test set, as commonly implemented, is used to set an expectation of performance when applying the trained model to unseen data. Finally, the development set is to select the preferred model instance. In an OOD problem setting, data are split (see Table 1) such that development set is an OOD with respect to the train set, and similarly the test set is an OOD from both the train and development sets. The deployment gap is calculated by deducting OOD-dev performance from the OOD-test performance.

#### Statistical model

The false positive rate (p-value) of predictions can be fitted into an extreme value distribution of the prediction scores (*R*^2^ = 0.98, *p*-*value* = 2.1*e*-5):

p-value=exp(-exp(21.7678x-11.0939))

where *x* is the raw prediction score of PortalCG.

E-value was estimated by *p*-*value* × 2.0 × 10^10^ for the chemical genomics space that includes the order of 10^6^ chemicals and approximate 20 thousands of human proteins.

### Baseline models

Machine learning methods for CPI predictions have been widely explored using many paradigms and approaches. As summarized in the survey [61], in addition to deep learning methods, there are similarity/distance-based methods, matrix factorization, network-based, and feature-based methods. For CPI predictions with the OOD generalization challenge, the similarity/distance-based, matrix factorization, and network-based methods have major obstacles. Similarity/distance-based methods rely on a drug-drug similarity matrix and a target-target similarity matrix as input. Because the similarities between dark proteins and proteins with known ligands are low, no reliable predictions can be made. Matrix Factorization is popular for its high efficiency, but the cold-start nature of dark proteins makes these less amenable to the matrix factorization paradigm. Network-based methods usually utilize protein-protein interactions. Such methods have advantages such as predicting the functional associations of ligand binding, but not the direct physical interactions. Furthermore, these methods are not scalable to millions of proteins and millions of chemicals. Almost all studies based on these approaches focus only on thousands of targets and thousands of drugs. PortalCG belongs to a category of feature-based approaches. In recently published work [1], we showed that DISAE outperforms other state-of-the-art feature-based methods; therefore,we primarily compared PortalCG with DISAE in the present paper.

Besides machine learning methods, protein-ligand docking (PLD) is a widely used approach to predict CPIs. We evaluated the performance of PLD, performed by (i) using AutoDock Vina [32] with (ii) 3D structures that were either experimentally determined or, in some cases, AlphaFold2-predicted [5], and (iii) followed by SIGN re-scoring ([33]; the Structure-aware Interactive Graph Neural Networks (SIGN) [33] method is a graph neural network for the prediction of protein-ligand binding affinity). SIGN builds directional graphs to model the structures and interactions in protein-ligand complexes. Both distances and angles are integrated in the aggregation processes. SIGN is trained on PDBbind [62], which is a well-known public dataset containing 3D structures of protein-ligand complexes together with experimentally determined binding affinities. Similar to what was done in SIGN [33], we used the PDBbind v2016 dataset and the corresponding refined set, which contains 3767 complexes, to perform training. We followed SIGN [33] for training and testing. For the directional graph used in SIGN, we constructed them with cutoff-threshold *θ*_*d*_ = 5Å. The number of hidden layers is set to 2. All of the other settings are kept the same as those used in the original paper of SIGN. We randomly split the PDBbind refined set with a ratio of 9:1 for training and validation.

## Supporting information

S1 Text Supportinng information of methods. More details on implementation, evaluation metrics, docking methods, compound desgin and synthesis and additional results in teh dark chemical genomics sequence exploration. **Table A: Model architecture configuration. Table B: 65 compounds tested for selective dual DRD1/3 antagonists. Table C: Undruggable human disease-associated proteins selected by ProtalCG. Table D: Predicted ligands for the transcription factors and transcription activity related proteins. Table E: Chemicals interacted with undruggable human proteins. Table F: Targets predicted by PortalCG for AI-10–49, fenebrutinib, PF-05190457 and their docking score from Autodock Vina. Table G: Functional Annotation enrichment for human proteins in Tbio selected by PortalCG. Table H: Chemicals interacted with human proteins in Tbio. Table I: Functional Annotation enrichment for undruggable human disease proteins without Tbio selected by PortalCG. Table J: Top ranked diseases associated with undruggable human proteins excluding Tbio selected by PortalCG. Table K: Chemicals interacted with undruggable human proteins excluding Tbio. Fig A: Model performance breakdown to each class**. In the main text, overall evaluation across positive and negative classes are reported, such as F1, ROC-AUC, PR-AUC. Here is a breakdown of performance in each class, where class0 is negative, i.e. not binding, class1 is positive, i.e. binding. against DISAE as baseline. **Fig B: Performance comparison of PLD+SIGN and Autodock Vina** Performance comparison of PLD+SIGN and Autodock Vina using the same OOD-test set as in main text: ROC-AUCs of Autodock Vina and PLD+SIGN are 0.535 and 0.569, respectively. PR-AUCs of Autodock Vina and PLD+SIGN 0.398 and 0.433, respectively. **Fig C: t-test comparison**. t-test comparison. The p-values for both ROC-AUC and PR-AUC are close to 0 against DISAE as baseline. **Fig D: Stress model selection performance curves against DISAE as baselinE. Fig E: Ratio 1:50 for DUD.E**. The ratio of inactive CPIs vs active CPIs under different Tanimoto coefficients of chemical similarities in the training data. The ratio of total inactive CPIs vs active CPIs is 1:1. **Fig F: Histogram equalization results** The left panel shows the original distribution of distance real values; to formalize a multi-class classification where each class has equal probability, histogram equalization transforms the distribution to the right panel of 10 bins, each as a class.
(PDF)
