# Supplementary material for: End-to-end sequence-structure-function meta-learning predicts genome-wide chemical-protein interactions for dark proteins
Source: PLoS Comput Biol. 2023 Jan 18;19(1):e1010851. doi: 10.1371/journal.pcbi.1010851 (PMC9886305; doi:10.1371/journal.pcbi.1010851)
Supplement: S1 Text — More details on implementation, evaluation metrics, docking methods, compound desgin and synthesis and additional results in teh dark chemical genomics sequence exploration. Table A: Model architecture configuration. Table B: 65 compounds tested for selective dual DRD1/3 antagonists. Table C: Undruggable human disease-associated proteins selected by ProtalCG. Table D: Predicted ligands for the transcription factors and transcription activity related proteins. Table E: Chemicals interacted with undruggable human proteins. Table F: Targets predicted by PortalCG for AI-10–49, fenebrutinib, PF-05190457 and their docking score from Autodock Vina. Table G: Functional Annotation enrichment for human proteins in Tbio selected by PortalCG. Table H: Chemicals interacted with human proteins in Tbio. Table I: Functional Annotation enrichment for undruggable human disease proteins without Tbio selected by PortalCG. Table J: Top ranked diseases associated with undruggable human proteins excluding Tbio selected by PortalCG. Table K: Chemicals interacted with undruggable human proteins excluding Tbio. Fig A: Model performance breakdown to each class. In the main text, overall evaluation across positive and negative classes are reported, such as F1, ROC-AUC, PR-AUC. Here is a breakdown of performance in each class, where class0 is negative, i.e. not binding, class1 is positive, i.e. binding. against DISAE as baseline. Fig B: Performance comparison of PLD+SIGN and Autodock Vina Performance comparison of PLD+SIGN and Autodock Vina using the same OOD-test set as in main text: ROC-AUCs of Autodock Vina and PLD+SIGN are 0.535 and 0.569, respectively. PR-AUCs of Autodock Vina and PLD+SIGN 0.398 and 0.433, respectively. Fig C: t-test comparison. t-test comparison. The p-values for both ROC-AUC and PR-AUC are close to 0 against DISAE as baseline. Fig D: Stress model selection performance curves against DISAE as baselinE. Fig E: Ratio 1:50 for DUD.E. The ratio of inactive CPIs vs active CPIs [file pcbi.1010851.s001.pdf]

# - **Supplemental Materials** -

End-to-end Sequence-structure-function Meta-learning Predicts Genome-Wide Chemical-Protein Interactions for Dark Proteins

Tian Cai[1], Li Xie[2], Shuo Zhang[1], Muge Chen[3], Di He[1], Amitesh Badkul[2], Yang Liu[2], Hari Krishna Namballa[4], Michael Dorogan[4], Wayne W. Harding[4], Cameron Mura[5], Philip E. Bourne[5], and Lei Xie[1,2,6,*]

[1]*Ph.D. Program in Computer Science, The Graduate Center, The City University of New York, New York, 10016, USA*

[2]*Department of Computer Science, Hunter College, The City University of New York, New York, 10065, USA*

[3]*Master Program in Computer Science, Courant Institute of Mathematical Sciences, New York University*

[4]*Department of Chemistry, Hunter College, The City University of New York, New York, 10065, USA*

[5]*School of Data Science & Department of Biomedical Engineering, University of Virginia, Virginia, 22903, USA*

[6]*Helen and Robert Appel Alzheimer's Disease Research Institute, Feil Family Brain & Mind Research Institute, Weill Cornell Medicine, Cornell University, New York, 10021, USA*

[*]*lei.xie@hunter.cuny.edu*

Contents

1 Methods **3**

1.1 Implementation details . . . 3

1.2 Evaluation metrics . . . 3

1.3 Docking as baseline . . . 3

1.4 Production level for deployment . . . 3

1.5 Additional results in dark chemical genomics space exploration . . . 3

1.6 Compound design and synthesis . . . 4

1.6.1 Serial 1 compounds . . . 4

1.6.2 Serial 2 compounds . . . 5

1.6.3 Serial 3 compounds . . . 5

1.6.4 Serial 4 compounds . . . 5

1.6.5 Serial 5 compounds . . . 5

2 Additional tables **8**

Table A in S1 Text: Model architecture configuration . . . 8

Table B in S1 Text: 65 compounds tested for selective dual DRD1/3 antagonists. . . . 8

Table C in S1 Text : Undruggable human disease-associated proteins selected by ProtalCG 10

Table D in S1 Text : Predicted ligands for the transcription factors and transcription activity related proteins. . . . 18

Table E in S1 Text: Chemicals interacted with undruggable human proteins . . . 18

Table F in S1 Text: Targets predicted by PortalCG for AI-10-49, fenebrutinib, PF-05190457 and their docking score from Autodock Vina. 18

Table G in S1 Text: Functional Annotation enrichment for human proteins in Tbio selected by PortalCG . . . 20

Table H in S1 Text : Chemicals interacted with human proteins in Tbio . . . 20

Table I in S1 Text: Functional Annotation enrichment for undruggable human disease proteins without Tbio selected by PortalCG . . . 20

Table J in S1 Text: Top ranked diseases associated with undruggable human proteins excluding Tbio selected by PortalCG . . . 20

Table K in S1 Text: Chemicals interacted with undruggable human proteins excluding Tbio 20

3 Additional figures **23**

Fig A in S1 Text: Model performance breakdown to each class . . . 23

Fig B in S1 Text: Performance comparison of PLD+SIGN and Autodock Vina . . . 23

Fig C in S1 Text: t-test comparison . . . 23

Fig D in S1 Text: Stress model selection performance curves against DISAE as baseline . 23

Fig E in S1 Text: Ratio 1:50 for DUD.E . . . 23

Fig F in S1 Text: Histogram equalization results . . . 23

Reference **28**

1 Methods

In this section, we present the detailed methodology of PortalCG.

1.1 Implementation details

Four major variants of models are trained as shown in main content Table 3 for controlled factor experiments to verify the contribution of key components of PortalCG. In this section we present implementation details.

Due to the large number of total samples, all training are carried out under global step-based formalization instead of epoch-based. Typically, a deep learning model is trained for numerous epochs, in each epoch the model will loop over all training data. Evaluation will be carried out once on the whole test data set at the end of each epoch. In the global step formalization, a mini-batch is sampled at uniform random from pre-split training data set. For a pre-defined total number of global steps, this mini-batch sampling will be repeated. To evaluate along the way of training, for every m global steps of training, a subset of OOD-test data is sampled uniformly randomly from a pre-split test set. To compute generalization gaps, in addition to evaluate on OOD-test set split according to the shifted evaluation, an OOD-dev set and and IID-dev are held out comparing to the train set distribution for the evaluation as well.

1.2 Evaluation metrics

Distogram prediction uses an average accuracy on the distogram. CPI binary classification uses F1, ROC-AUC and PR-AUC for overall evaluation with breakdown by class F1, recall and precision scores.

1.3 Docking as baseline

Protein-ligand docking was performed using Autodock Vina[1]. In the benchmark study, the whole protein surface search implemented in the Autodock Vina was applied to identify the ligand binding pocket. The center of each protein was set as the center of the binding pocket. For the DRD1/DRD2/DRD3, the center of the co-crystallized ligand was set as the center of the binding pocket. The largest distance of the protein atoms to the center of the protein is calculated for each x, y, and z direction to define the edge of each protein. 10 Angstrom of extra space was added to the protein edge to set up the search space for the docking. We selected the 9 binding poses that had the best scores.

1.4 Production level for deployment

To create a production level model, three models were trained in PortalCG with only difference in data split. Dev set was OOD in respect of training set to make sure there was no overlapped Pfam families between them. By rotating Pfam families between training set and OOD-dev set in the fashion of a cross-validation, each of the three models was trained on different train set in light of Pfam families involved. Then a voting mechanism was used to make the final prediction.

1.5 Additional results in dark chemical genomics space exploration

When we consider the proteins in Tbio, there are 9545 proteins which are not int Casas's druggable proteins. If 0.67 was used as the cutoff, 219 proteins were predicted as positive hits. The gene enrichment analysis result for these proteins was listed in Table G in S1 Text. Disease associated

with these 219 human proteins were also listed in main context Table 5. Since one protein is always related with multiple diseases, these diseases are ranked by the number of their associated proteins and the top 10 diseases were listed in the table. Most of top ranked diseases are related with cancer development. 21 drugs that are approved or in clinical trial are predicted to interact with these proteins as shown in Table H in S1 Text.

If the proteins in Tbio were removed from the undruggable list, only 2930 proteins were left. If 0.67 was used as the cutoff, there will be only 41 proteins predicted positive and no significant enrichment with David gene enrichment analysis. So 0.665 was used as a cutoff, and 348 proteins were predicted as positive hits. The gene enrichment analysis result for these proteins was listed in Table I in S1 Text. Disease associated with these 348 human proteins were also listed in Table J in S1 Text. 42 drugs that are approved or in clinical trial are predicted to interact with these proteins as shown in Table K in S1 Text.

1.6 Compound design and synthesis

5 series of compounds were designed and synthesized as detailed below.

1.6.1 Serial 1 compounds

Serial 1 compounds were synthesized as shown in Scheme 1 and as described in reference [2]. Briefly, the brominated phenol tetrahydroprotoberberine derivative 1 was alkylated with various alkyl halides to install the C9 alkoxy groups in compounds 2a-2g. Debenzylation of compounds 2a-2g via treatment with ethanolic HCl afforded analogs 3a-3g. Removal of the bromine group in compounds 2a-2g gave compounds 4a-4g, which were subsequently debenzylated to afford analogs 5a-5g.

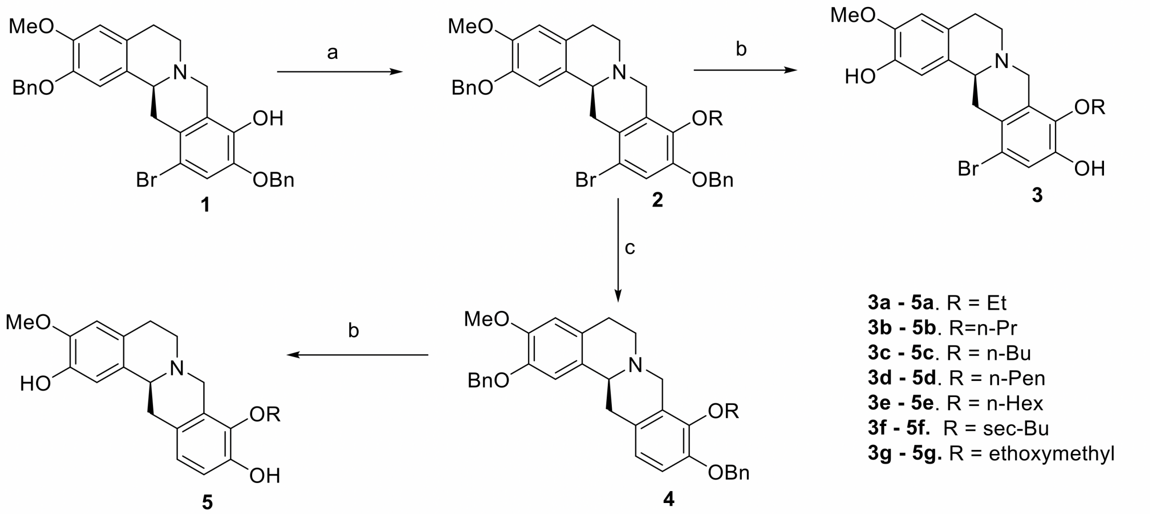

Scheme 1. *Reagents and condition:* a) appropriate alkyl halide, K_2CO_3, DMF, 2-4h; b) conc. HCl, EtOH, 70 °C, 1.5 – 2 h; c) i-PrMgCl, LiCl, THF, 0 °C, 2 h

1.6.2 Serial 2 compounds

The synthesis of serial 2 compounds is shown in Scheme 2 and is described in detail in reference [?]. Briefly, the benzylated tetrahydroisoquinoline 6 was debenzylated to give compound 7. Treatment of the phenol 6 with appropriate alkyl halides allowed for alkylation of the C10 phenol group of 6. The intermediate C10 alkoxy derivatives were then debenzylated to afford the C10 alkoxylated/C2 phenol derivatives 8a – 8h.

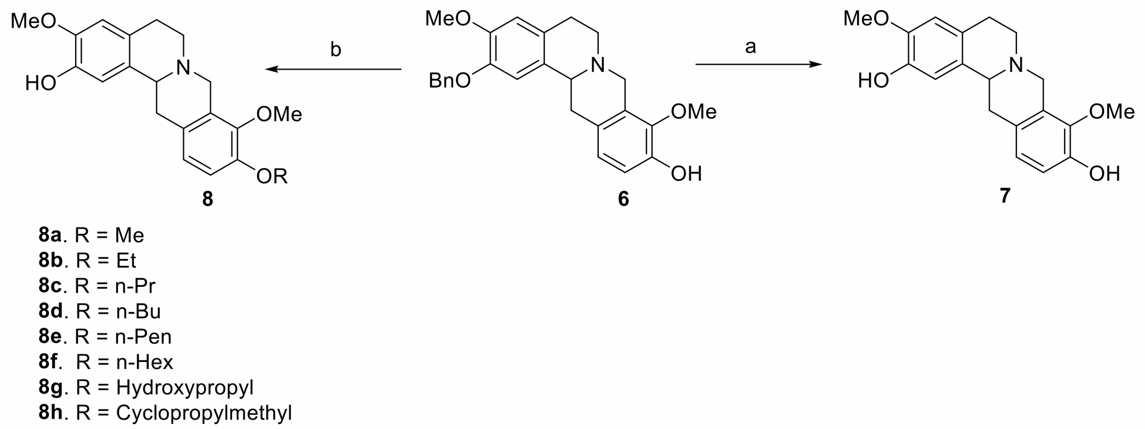

Scheme 2. *Reagents and conditions*: conc. HCl, MeOH, reflux; b) (i) appropriate akyl halide, DMF, CH_3CN, rt to reflux; (ii) conc. HCl, MeOH, reflux.

1.6.3 Serial 3 compounds

Serial 3 ompounds were synthesized as depicted in Scheme 3. Further details on the synthesis of the analogs and precursors may be found in reference [3]. The acetate group in compound 9 was hydrolyzed to an intermediate alcohol, which was subsequently reacted with thionyl chloride. The alkyl chloride thus formed underwent ring closure to form tetrahydroprotoberberine 10. The C3 phenol of 10 was methylated and the C2 and C10 benzyl groups of the resulting compounds were removed under acidic conditions to afford analogs 11a-11e.

1.6.4 Serial 4 compounds

Synthesis of the serial 4 compounds is shown in Scheme 4 and reported in a previous publication [4]. The amine 12 was coupled to various carboxylic acids and the resulting benzylated amides were debenzylated by refluxing in ethanolic HCl, yielding analogs 13a-13m. The phenol 13m was methylated to provide analog 13n.

1.6.5 Serial 5 compounds

Serial 5 compounds were synthesized as depicted in Scheme 5 and as previously reported in reference [5]. Briefly, amine 14a was coupled with various aromatic carboxylic acids to give intermediate amides which were subsequently debenzylated to give the corresponding amide analogs 15a-15r.

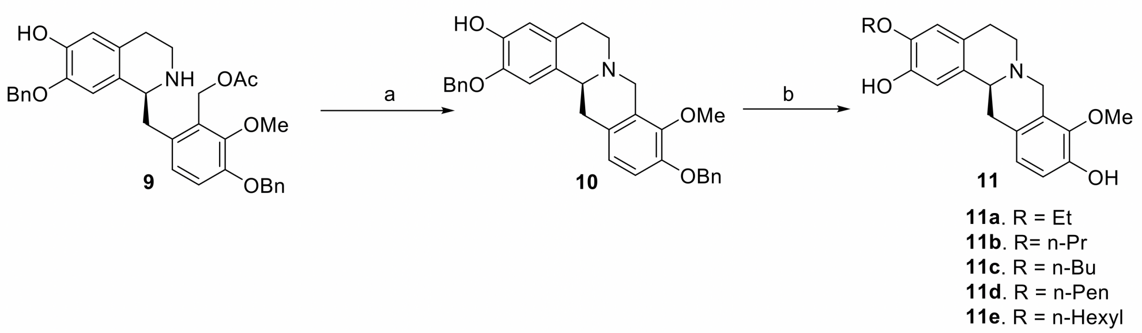

Scheme 3. *Reagents and conditions*: a) (i) 10% NaOH, EtOH, rt; (ii) $SOCl_2$, CH_2Cl_2, $NaHCO_3$; b) RBr, K_2CO_3, DMF, rt; (ii) conc. HCl, EtOH, reflux

Compounds 15s-15t were synthesized by amide coupling of amine 14b with the corresponding cyanophenyl carboxylic acids. Compounds 16a-16c were synthesized by treatment of corresponding precursors in the compound 15 series with BBr3, to effect demethylation to the catechol functionality.

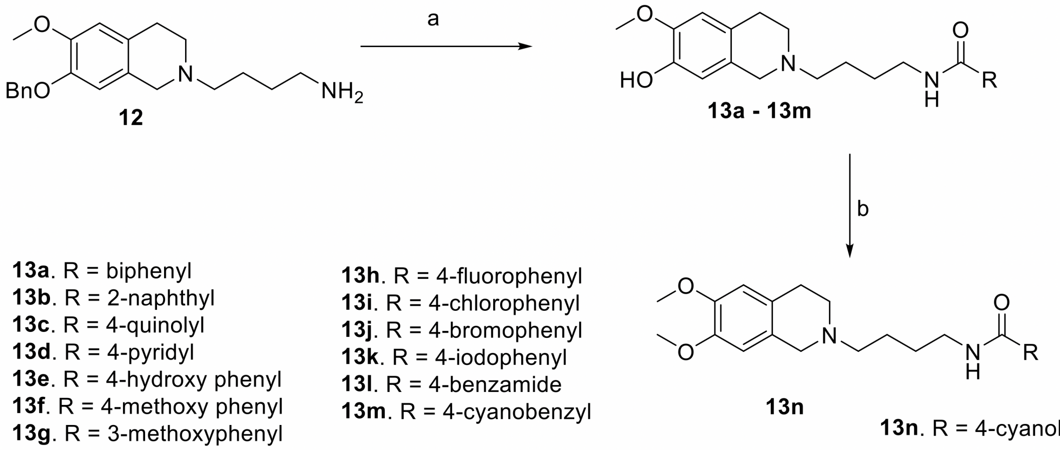

13a. R = biphenyl
13b. R = 2-naphthyl
13c. R = 4-quinolyl
13d. R = 4-pyridyl
13e. R = 4-hydroxy phenyl
13f. R = 4-methoxy phenyl
13g. R = 3-methoxyphenyl
13h. R = 4-fluorophenyl
13i. R = 4-chlorophenyl
13j. R = 4-bromophenyl
13k. R = 4-iodophenyl
13l. R = 4-benzamide
13m. R = 4-cyanobenzyl

13n. R = 4-cyanobenzyl

Scheme 4. *Reagents and conditions*: a) (i)RCOOH, EDC, DCM, rt; (ii) Conc. HCl, EtOH, reflux; b) **13m**, MeI, K_2CO_3, acetone

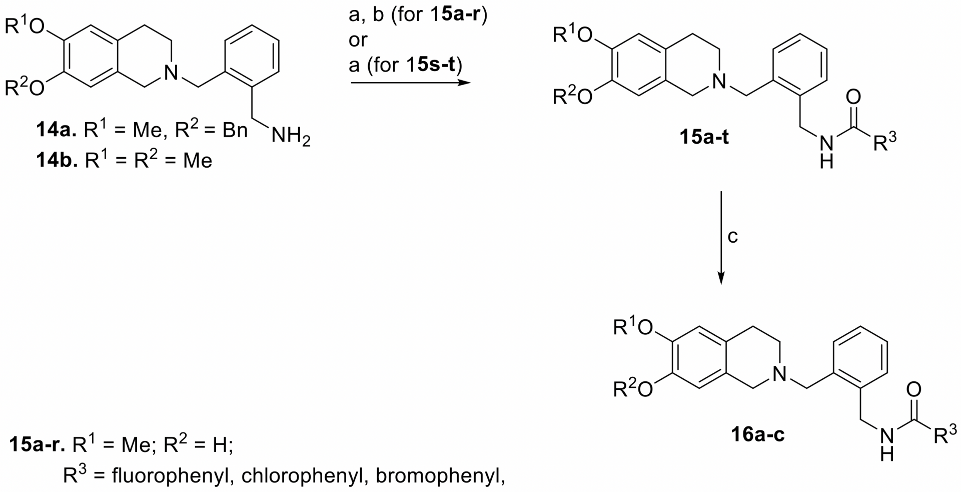

15a-r. R^1 = Me; R^2 = H;
R^3 = fluorophenyl, chlorophenyl, bromophenyl, iodophenyl, methoxyphenyl, cyanophenyl, 2-naphthyl, biphenyl
15s. R^1 = R^2 = Me; R^3 = 3-cyanophenyl
15t. R^1 = R^2 = Me; R^3 = 4-cyanophenyl

16a. R^1 = R^2 = H; R^3 = 3-bromophenyl
16b. R^1 = R^2 = H; R^3 = 3-cyanophenyl
16c. R^1 = R^2 = H; R^3 = 4-cyanophenyl

Scheme 5. *Reagents and conditions*: (a) HBTU, appropriate carboxylic acid, TEA, 12 h, rt; (b) conc. HCl, CH_3COOH, 12 h, 40 °C; (c) BBr_3, DCM, 0°C, 2h

2 Additional tables

Table A in S1 Text: Model architecture configuration

Protein descriptor	layers	Albert –>Resnet
	embedding dimension	256
Chemical descriptor	backbone	GIN
	number of layers	5
	embedding dimension	300
	aggregation methods	sum
	drop out ratio	0.5
Interaction learner	layers	Attentive pooling –>2 layers of MLP
	embedding dimension	128
Structure residue-atom pair wise feature learner	layers	matrix multiplication of protein and chemical embedding vectors
Classifier	layers	2 layers of MLP
	embedding dimension	64

Table B in S1 Text: 65 compounds tested for selective dual DRD1/3 antagonists. na: no detectable binding affinity.

SMILES	D1R Ki in nM	D3R Ki in nM	D2R Ki in nM
BrC1=CC(O)=C(OCC)C2=C1C[C@H]3C4=CC(O)=C(OC)C=C4CCN3C2	1.3	373	na
BrC1=CC(O)=C(OCCC)C2=C1C[C@H]3C4=CC(O)=C(OC)C=C4CCN3C2	2.2	195	70
BrC1=CC(O)=C(OCCCC)C2=C1C[C@H]3C4=CC(O)=C(OC)C=C4CCN3C2	2.3	na	107
BrC1=CC(O)=C(OCCCCC)C2=C1C[C@H]3C4=CC(O)=C(OC)C=C4CCN3C2	32	3605	398
BrC1=CC(O)=C(OCCCCCC)C2=C1C[C@H]3C4=CC(O)=C(OC)C=C4CCN3C2	68	3171	731
BrC1=CC(O)=C(OCC(C)C)C2=C1C[C@H]3C4=CC(O)=C(OC)C=C4CCN3C2	9.7	2070	331
BrC1=CC(O)=C(OCCOC)C2=C1C[C@H]3C4=CC(O)=C(OC)C=C4CCN3C2	4.2	726	na
OC1=C(OC)C=C2C([C@H](CC(C=CC(O)=C3OCC)=C3C4)N4CC2)=C1	5.3	106	na
OC1=C(OC)C=C2C([C@H](CC(C=CC(O)=C3OCCC)=C3C4)N4CC2)=C1	12	175	41
OC1=C(OC)C=C2C([C@H](CC(C=CC(O)=C3OCCCC)=C3C4)N4CC2)=C1	11	131	na
OC1=C(OC)C=C2C([C@H](CC(C=CC(O)=C3OCCCCC)=C3C4)N4CC2)=C1	31	3213	23
OC1=C(OC)C=C2C([C@H](CC(C=CC(O)=C3OCCCCCC)=C3C4)N4CC2)=C1	77	195	na
OC1=C(OC)C=C2C([C@H](CC(C=CC(O)=C3OCC(C)C)=C3C4)N4CC2)=C1	65	450	104
OC1=C(OC)C=C2C([C@H](CC(C=CC(O)=C3OCCOC)=C3C4)N4CC2)=C1	7.7	31	18
OC1=C(OC)C=C2C([C@H](CC(C=CC(O)=C3OC)=C3C4)N4CC2)=C1	5.9	30	974
OC1=C(OC)C=C2C([C@H](CC(C=CC(OC)=C3OC)=C3C4)N4CC2)=C1	15	37	102
OC1=C(OC)C=C2C([C@H](CC(C=CC(OCC)=C3OC)=C3C4)N4CC2)=C1	124	44	159
OC1=C(OC)C=C2C([C@H](CC(C=CC(OCCC)=C3OC)=C3C4)N4CC2)=C1	7.8	53	99
OC1=C(OC)C=C2C([C@H](CC(C=CC(OCCCC)=C3OC)=C3C4)N4CC2)=C1	11	65	121
OC1=C(OC)C=C2C([C@H](CC(C=CC(OCCCCC)=C3OC)=C3C4)N4CC2)=C1	19	101	283
OC1=C(OC)C=C2C([C@H](CC(C=CC(OCCCCCC)=C3OC)=C3C4)N4CC2)=C1	40	220	na
OC1=C(OC)C=C2C([C@H](CC(C=CC(OCCCO)=C3OC)=C3C4)N4CC2)=C1	6.7	110	211
OC1=C(OC)C=C2C([C@H](CC(C=CC(OCC3CC3)=C4OC)=C4C5)N5CC2)=C1	12	54	54
OC1=C(OCC)C=C2C([C@H](CC(C=CC(O)=C3OC)=C3C4)N4CC2)=C1	17	40	2089
OC1=C(OCCC)C=C2C([C@H](CC(C=CC(O)=C3OC)=C3C4)N4CC2)=C1	15	46	2682
OC1=C(OCCCC)C=C2C([C@H](CC(C=CC(O)=C3OC)=C3C4)N4CC2)=C1	53	51	2739
OC1=C(OCCCCC)C=C2C([C@H](CC(C=CC(O)=C3OC)=C3C4)N4CC2)=C1	31	33	1433
OC1=C(OCCCCCC)C=C2C([C@H](CC(C=CC(O)=C3OC)=C3C4)N4CC2)=C1	42	26	1491
OC1=C(C=C2CCN(CC2=C1)CCCCNC(C3=CC=C(C4=CC=CC=C4)C=C3)=O)OC	800	2.7	800
OC1=C(C=C2CCN(CC2=C1)CCCCNC(C3=CC=C(C=CC=C4)C4=C3)=O)OC	800	2.7	700
OC1=C(C=C2CCN(CC2=C1)CCCCNC(C3=CC=NC4=C3C=CC=C4)=O)OC	1810	22	na
OC1=C(C=C2CCN(CC2=C1)CCCCNC(C3=CC=NC=C3)=O)OC	na	24	na
OC1=C(C=C2CCN(CC2=C1)CCCCNC(C3=CC=C(O)C=C3)=O)OC	na	8.7	na
OC1=C(C=C2CCN(CC2=C1)CCCCNC(C3=CC=C(OC)C=C3)=O)OC	na	5.9	na
OC1=C(C=C2CCN(CC2=C1)CCCCNC(C3=CC=C(F)C=C3)=O)OC	na	4.4	na
OC1=C(C=C2CCN(CC2=C1)CCCCNC(C3=CC=C(Cl)C=C3)=O)OC	680	2.1	1100
OC1=C(C=C2CCN(CC2=C1)CCCCNC(C3=CC=C(Br)C=C3)=O)OC	500	3.4	na
OC1=C(C=C2CCN(CC2=C1)CCCCNC(C3=CC=C(I)C=C3)=O)OC	1600	10	na
OC1=C(C=C2CCN(CC2=C1)CCCCNC(C3=CC=C(C(N)=O)C=C3)=O)OC	na	28	860
OC1=C(C=C2CCN(CC2=C1)CCCCNC(C3=CC=C(C#N)C=C3)=O)OC	na	6.3	na
OC1=C(C=C2CCN(CC2=C1)CCCCNC(C3=CC=CC(OC)=C3)=O)OC	na	12	na
O=C(C1=CC=C(C#N)C=C1)NCCCCN(CC2=C3)CCC2=CC(OC)=C3OC	na	410	na
OC1=C(C=C2CCN(CC2=C1)CC3=C(CNC(C4=CC=C(C5=CC=CC=C5)C=C4)=O)C=CC=C3)OC	na	na	na
OC1=C(C=C2CCN(CC2=C1)CC3=C(CNC(C4=CC=C(C=CC=C5)C5=C4)=O)C=CC=C3)OC	na	780	310
OC1=C(C=C2CCN(CC2=C1)CC3=C(CNC(C4=CC=CC=C4Cl)=O)C=CC=C3)OC	na	380	190
OC1=C(C=C2CCN(CC2=C1)CC3=C(CNC(C4=CC=CC=C4Br)=O)C=CC=C3)OC	na	540	420
OC1=C(C=C2CCN(CC2=C1)CC3=C(CNC(C4=CC=CC=C4OC)=O)C=CC=C3)OC	na	120	78
OC1=C(C=C2CCN(CC2=C1)CC3=C(CNC(C4=CC=CC(Cl)=C4)=O)C=CC=C3)OC	na	140	100
OC1=C(C=C2CCN(CC2=C1)CC3=C(CNC(C4=CC=CC(Br)=C4)=O)C=CC=C3)OC	na	170	150
OC1=C(C=C2CCN(CC2=C1)CC3=C(CNC(C4=CC=CC(OC)=C4)=O)C=CC=C3)OC	na	280	140
OC1=C(C=C2CCN(CC2=C1)CC3=C(CNC(C4=CC=CC(C#N)=C4)=O)C=CC=C3)OC	na	26	39
OC1=C(C=C2CCN(CC2=C1)CC3=C(CNC(C4=CC=C(F)C=C4)=O)C=CC=C3)OC	na	140	52
OC1=C(C=C2CCN(CC2=C1)CC3=C(CNC(C4=CC=C(Cl)C=C4)=O)C=CC=C3)OC	na	55	47
OC1=C(C=C2CCN(CC2=C1)CC3=C(CNC(C4=CC=C(Br)C=C4)=O)C=CC=C3)OC	na	160	76
OC1=C(C=C2CCN(CC2=C1)CC3=C(CNC(C4=CC=C(I)C=C4)=O)C=CC=C3)OC	na	na	84
OC1=C(C=C2CCN(CC2=C1)CC3=C(CNC(C4=CC=C(OC)C=C4)=O)C=CC=C3)OC	na	180	74
OC1=C(C=C2CCN(CC2=C1)CC3=C(CNC(C4=CC=C(C#N)C=C4)=O)C=CC=C3)OC	na	310	190
OC1=C(C=C2CCN(CC2=C1)CC3=C(CNC(C4=CC=CC(Cl)=C4Cl)=O)C=CC=C3)OC	na	460	280
OC1=C(C=C2CCN(CC2=C1)CC3=C(CNC(C4=CC=CC(OC)=C4OC)=O)C=CC=C3)OC	na	57	na
OC1=C(C=C2CCN(CC2=C1)CC3=C(CNC(C4=CC=C(I)C(OC)=C4)=O)C=CC=C3)OC	1970	24	27
O=C(C1=CC=CC(C#N)=C1)NCC(C=CC=C2)=C2CN(CC3=C4)CCC3=CC(OC)=C4OC	510	1.2	46
O=C(C1=CC=C(C#N)C=C1)NCC(C=CC=C2)=C2CN(CC3=C4)CCC3=CC(OC)=C4OC	58	3.4	50
OC(C=C1CCN(CC1=C2)CC3=C(CNC(C4=CC=C(C#N)C=C4)=O)C=CC=C3)=C2O	na	28	83
OC(C=C1CCN(CC1=C2)CC3=C(CNC(C4=CC=CC(C#N)=C4)=O)C=CC=C3)=C2O	na	370	900
OC(C=C1CCN(CC1=C2)CC3=C(CNC(C4=CC=CC(Br)=C4)=O)C=CC=C3)=C2O	91	2	56

Table C in S1 Text: Undruggable human disease-associated proteins selected by ProtalCG.

Uniprot	Protein name	Drug name	e-value
Q8TEX9	Importin-4	fenebrutinib	1.75E-08
Q8NB66	Protein unc-13 homolog C	AI-10-49	5.96E-08
Q9NZF1	Placenta-specific gene 8 protein	AI-10-49	2.52E-07
Q96G03	Phosphoglucomutase-2	fenebrutinib	3.60E-07
Q8IWU4	Zinc transporter 8	fenebrutinib	3.60E-07
P53004	Biliverdin reductase A	AI-10-49	5.29E-07
P40879	Chloride anion exchanger	fenebrutinib	5.65E-07
Q8IYL2	Probable tRNA	fenebrutinib	5.65E-07
Q9ULL4	Plexin-B3	AI-10-49	7.59E-07
Q96SZ6	Mitochondrial tRNA methylthiotransferase CDK5RAP1	fenebrutinib	8.67E-07
Q9BRT9	DNA replication complex GINS protein SLD5	fenebrutinib	8.67E-07
Q8N2U0	Transmembrane protein 256	CCT137690	8.79E-07
Q9UC06	Zinc finger protein 70	fenebrutinib	1.06E-06
Q9BPX5	Actin-related protein 2/3 complex subunit 5-like protein	Q-203	1.47E-06
Q8TDF6	RAS guanyl-releasing protein 4	NMS-1286937	1.59E-06
Q9P2G3	Kelch-like protein 14	NMS-1286937	1.59E-06
Q9HBT7	Zinc finger protein 287	CCT137690	2.22E-06
Q9UKR8	Tetraspanin-16	PF-05190457	2.38E-06
P59044	NACHT, LRR and PYD domains-containing protein 6	MK-5046	2.65E-06
Q16774	Guanylate kinase	MK-5046	2.65E-06
Q9HCE5	N6-adenosine-methyltransferase non-catalytic subunit	PF-05190457	2.67E-06
Q15185	Prostaglandin E synthase 3	cilofexor	3.63E-06
Q8WUA7	TBC1 domain family member 22A	fenebrutinib	4.22E-06
Q17RS7	Flap endonuclease GEN homolog 1	CGM097	4.38E-06
Q14244	Ensconsin	PF-05190457	4.50E-06
Q9BRK5	45 kDa calcium-binding protein	PF-05190457	4.50E-06
Q9UKJ5	Cysteine-rich hydrophobic domain-containing protein 2	CCT137690	4.54E-06
Q6ZWJ1	Syntaxin-binding protein 4	AI-10-49	4.74E-06
Q9HAR2	Adhesion G protein-coupled receptor L3	AI-10-49	5.14E-06
P98161	Polycystin-1	AI-10-49	5.14E-06
Q92673	Sortilin-related receptor	AI-10-49	5.43E-06
D6RGH6	Multicilin	fenebrutinib	5.84E-06
Q8NHH9	Atlastin-2	fenebrutinib	5.93E-06
Q9H0E2	Toll-interacting protein	PF-05190457	6.17E-06
O15145	Actin-related protein 2/3 complex subunit 3	PF-05190457	6.17E-06
P51116	Fragile X mental retardation syndrome-related protein 2	abemaciclib	6.53E-06

Q9BR09	Neuralized-like protein 2	elbasvir	7.25E-06
P42568	Protein AF-9	AI-10-49	7.54E-06
P17600	Synapsin-1	AI-10-49	7.54E-06
P48553	Trafficking protein particle complex subunit 10	AI-10-49	7.77E-06
Q12955	Ankyrin-3	abemaciclib	8.42E-06
O60936	Nucleolar protein 3	abemaciclib	8.42E-06
Q02575	Helix-loop-helix protein 1	AI-10-49	8.47E-06
P49640	Homeobox even-skipped homolog protein 1	CFI-402257	8.56E-06
P22670	MHC class II regulatory factor RFX1	PF-05190457	8.66E-06
Q8IUF8	Ribosomal oxygenase 2	NMS-1286937	8.90E-06
P22681	E3 ubiquitin-protein ligase CBL	NMS-1286937	8.90E-06
Q7RTS3	Pancreas transcription factor 1 subunit alpha	MK-5046	9.50E-06
Q9Y5L4	Mitochondrial import inner membrane translocase subunit Tim13	NMS-P715	9.61E-06
P17735	Tyrosine aminotransferase	PF-05190457	1.03E-05
O95294	RasGAP-activating-like protein 1	PF-05190457	1.04E-05
Q8NCD3	Holliday junction recognition protein	PF-05190457	1.04E-05
Q86W28	NACHT, LRR and PYD domains-containing protein 8	MK-5046	1.10E-05
Q04671	P protein	AI-10-49	1.15E-05
Q9C035	Tripartite motif-containing protein 5	AI-10-49	1.15E-05
Q66K64	DDB1- and CUL4-associated factor 15	AI-10-49	1.29E-05
Q5T9A4	ATPase family AAA domain-containing protein 3B	CFI-402257	1.37E-05
Q96MP8	BTB/POZ domain-containing protein KCTD7	CFI-402257	1.37E-05
Q9UIE0	Zinc finger protein 230	CFI-402257	1.39E-05
Q3SYG4	Protein PTHB1	abemaciclib	1.44E-05
Q8IUY3	GRAM domain-containing protein 2A	AI-10-49	1.51E-05
O75689	Arf-GAP with dual PH domain-containing protein 1	AI-10-49	1.51E-05
Q9NZP8	Complement C1r subcomponent-like protein	fenebrutinib	1.55E-05
Q9NQX4	Unconventional myosin-Vc	fenebrutinib	1.55E-05
Q15388	Mitochondrial import receptor subunit TOM20 homolog	CFI-402257	1.57E-05
Q9NXL9	DNA helicase MCM9	piperaquine-phosphate	1.59E-05
P53370	Nucleoside diphosphate-linked moiety X motif 6	piperaquine-phosphate	1.59E-05
Q8N7C0	Leucine-rich repeat-containing protein 52	PF-05190457	1.65E-05
O95231	Homeobox protein VENTX	NMS-P715	1.66E-05
Q9HC56	Protocadherin-9	AI-10-49	1.71E-05
A6NNW6	Enolase 4	AI-10-49	1.71E-05

P35612	Beta-adducin	CFI-402257	1.74E-05
Q9Y6M5	Zinc transporter 1	AI-10-49	1.74E-05
Q01804	OTU domain-containing protein 4	AI-10-49	1.74E-05
Q9Y678	Coatomer subunit gamma-1	MK-5046	1.75E-05
Q9BTY7	Protein HGH1 homolog	AI-10-49	1.91E-05
Q8NEM0	Microcephalin	CFI-402257	1.91E-05
Q7LGA3	Heparan sulfate 2-O-sulfotransferase 1	AI-10-49	1.92E-05
O75956	Cyclin-dependent kinase 2-associated protein 2	ziritaxestat	1.97E-05
P62987	Ubiquitin-60S ribosomal protein L40	ziritaxestat	1.97E-05
Q14257	Reticulocalbin-2	CDK9-IN-6	1.97E-05
Q9H0I3	Coiled-coil domain-containing protein 113	tropifexor	2.03E-05
P32019	Type II inositol 1,4,5-trisphosphate 5-phosphatase	fenebrutinib	2.03E-05
O95409	Zinc finger protein ZIC 2	fenebrutinib	2.03E-05
P56179	Homeobox protein DLX-6	fenebrutinib	2.05E-05
Q12798	Centrin-1	AI-10-49	2.13E-05
Q14714	Sarcospan	AI-10-49	2.13E-05
Q96LI5	CCR4-NOT transcription complex subunit 6-like	abemaciclib	2.15E-05
Q86XR7	TIR domain-containing adapter molecule 2	AI-10-49	2.15E-05
P53672	Beta-crystallin A2	CCT137690	2.18E-05
O43439	Protein CBFA2T2	CCT137690	2.18E-05
Q9H0E3	Histone deacetylase complex subunit SAP130	fenebrutinib	2.19E-05
Q8NFZ0	F-box DNA helicase 1	fenebrutinib	2.19E-05
Q8N0S2	Synaptonemal complex central element protein 1	CFI-402257	2.21E-05
Q92667	A-kinase anchor protein 1, mitochondrial	fenebrutinib	2.22E-05
O60884	DnaJ homolog subfamily A member 2	PF-05190457	2.38E-05
P41214	Eukaryotic translation initiation factor 2D	AI-10-49	2.41E-05
Q6R327	Rapamycin-insensitive companion of mTOR	AI-10-49	2.42E-05
O75886	Signal transducing adapter molecule 2	PF-05190457	2.50E-05
Q9UMX6	Guanylyl cyclase-activating protein 2	PF-05190457	2.50E-05
Q99447	Ethanolamine-phosphate cytidylyltransferase	CCT137690	2.53E-05
Q6FI13	Histone H2A type 2-A	PF-05190457	2.59E-05
Q969S2	Endonuclease 8-like 2	fenebrutinib	2.62E-05
Q6XZF7	Dynamin-binding protein	AI-10-49	2.71E-05
Q96EX1	Small integral membrane protein 12	fenebrutinib	2.81E-05
Q8TD57	Dynein axonemal heavy chain 3	fenebrutinib	2.81E-05
O15375	Monocarboxylate transporter 6	PF-05190457	2.83E-05
Q9H7E2	Tudor domain-containing protein 3	NMS-1286937	2.86E-05

A6NI73	Leukocyte immunoglobulin-like receptor subfamily A member 5	AI-10-49	2.88E-05
Q9NZM3	Intersectin-2	CDK9-IN-6	2.94E-05
O75145	Liprin-alpha-3	CDK9-IN-6	2.94E-05
Q9NSD9	Phenylalanine–tRNA ligase beta subunit	abemaciclib	2.99E-05
O15195	Villin-like protein	abemaciclib	2.99E-05
P05538	HLA class II histocompatibility antigen, DQ beta 2 chain	cilofexor	3.01E-05
Q8TD57	Dynein axonemal heavy chain 3	cilofexor	3.01E-05
Q9NRD9	Dual oxidase 1	abemaciclib	3.04E-05
Q8WUQ7	Cactin	AI-10-49	3.10E-05
Q9H7C4	Syncoilin	AI-10-49	3.10E-05
Q70EK9	Ubiquitin carboxyl-terminal hydrolase 51	PF-05190457	3.19E-05
O43837	Isocitrate dehydrogenase [NAD] subunit beta, mitochondrial	PF-05190457	3.19E-05
Q9UKY1	Zinc fingers and homeoboxes protein 1	CFI-402257	3.20E-05
Q6ZMJ2	Scavenger receptor class A member 5	CFI-402257	3.20E-05
P61371	Insulin gene enhancer protein ISL-1	fenebrutinib	3.23E-05
Q6NSZ9	Zinc finger and SCAN domain-containing protein 25	fenebrutinib	3.23E-05
Q92754	Transcription factor AP-2 gamma	IACS-10759	3.26E-05
Q5RKV6	Exosome complex component MTR3	AI-10-49	3.31E-05
Q9BRA0	N-alpha-acetyltransferase 38, NatC auxiliary subunit	MK-5046	3.35E-05
Q9BSF8	BTB/POZ domain-containing protein 10	CCT137690	3.42E-05
Q6PJ69	Tripartite motif-containing protein 65	AI-10-49	3.45E-05
Q9NRP0	Oligosaccharyltransferase complex subunit OSTC	AI-10-49	3.45E-05
Q8TBM7	Transmembrane protein 254	PF-05190457	3.47E-05
Q674X7	Kazrin	elbasvir	3.54E-05
Q9Y3C4	EKC/KEOPS complex subunit TPRKB	abemaciclib	3.57E-05
P59190	Ras-related protein Rab-15	abemaciclib	3.57E-05
Q9NZM3	Intersectin-2	PF-05190457	3.69E-05
P05455	Lupus La protein	AI-10-49	3.80E-05
Q96KN3	Homeobox protein PKNOX2	abemaciclib	3.86E-05
P54278	Mismatch repair endonuclease PMS2	abemaciclib	3.86E-05
P49069	Guided entry of tail-anchored proteins factor CAMLG	fenebrutinib	3.98E-05
Q8IYT4	Katanin p60 ATPase-containing subunit A-like 2	AI-10-49	3.99E-05
Q8WVI0	Small integral membrane protein 4	AI-10-49	3.99E-05
Q9Y6X6	Unconventional myosin-XVI	MK-5046	4.23E-05
Q8N357	Solute carrier family 35 member F6	MK-5046	4.23E-05
Q96BP2	Coiled-coil-helix-coiled-coil-helix domain-containing protein 1	abemaciclib	4.24E-05
Q9UJQ4	Sal-like protein 4	MK-5046	4.26E-05

Q9HCQ5	Polypeptide N-acetylgalactosaminyltransferase 9	fenebrutinib	4.33E-05
Q9P2Q2	FERM domain-containing protein 4A	fenebrutinib	4.33E-05
Q7Z7M8	UDP-GlcNAc:betaGal beta-1,3-N-acetylglucosaminyltransferase 8	NMS-P715	4.37E-05
Q9HCK5	Protein argonaute-4	PF-05190457	4.42E-05
O95405	Zinc finger FYVE domain-containing protein 9	AI-10-49	4.50E-05
Q92911	Sodium/iodide cotransporter	PF-05190457	4.51E-05
Q8WWI1	LIM domain only protein 7	fenebrutinib	4.54E-05
Q8N3C7	CAP-Gly domain-containing linker protein 4	fenebrutinib	4.54E-05
Q9UBS8	E3 ubiquitin-protein ligase RNF14	PF-05190457	4.56E-05
Q9BY66	Lysine-specific demethylase 5D	PF-05190457	4.56E-05
Q9UPM8	AP-4 complex subunit epsilon-1	abemaciclib	4.72E-05
Q9BYE0	Transcription factor HES-7	MK-5046	4.91E-05
Q9BSD7	Cancer-related nucleoside-triphosphatase	AI-10-49	4.94E-05
Q9NXH9	tRNA	tropifexor	4.99E-05
Q13948	Protein CASP	tropifexor	4.99E-05
Q9C019	Tripartite motif-containing protein 15	abemaciclib	5.08E-05
Q5TA50	Ceramide-1-phosphate transfer protein	ABT-702	5.09E-05
Q7KZF4	Staphylococcal nuclease domain-containing protein 1	ABT-702	5.09E-05
P08247	Synaptophysin	fenebrutinib	5.09E-05
A4IF30	Solute carrier family 35 member F4	AI-10-49	5.13E-05
Q9NRA8	Eukaryotic translation initiation factor 4E transporter	AI-10-49	5.13E-05
Q96FF9	Sororin	acalabrutinib	5.16E-05
P10073	Zinc finger and SCAN domain-containing protein 22	PF-05190457	5.22E-05
P0CW19	LIM and senescent cell antigen-like-containing domain protein 3	PF-05190457	5.22E-05
Q9UPM8	AP-4 complex subunit epsilon-1	fenebrutinib	5.23E-05
P08118	Beta-microseminoprotein	PF-05190457	5.33E-05
Q96P16	Regulation of nuclear pre-mRNA domain-containing protein 1A	fenebrutinib	5.39E-05
P62280	40S ribosomal protein S11	piperaquine-phosphate	5.42E-05
Q8TE49	OTU domain-containing protein 7A	AI-10-49	5.43E-05
Q53HI1	Protein unc-50 homolog	AI-10-49	5.43E-05
Q8WW32	High mobility group protein B4	PF-05190457	5.54E-05
P57729	Ras-related protein Rab-38	PF-05190457	5.54E-05
Q8N2M8	CLK4-associating serine/arginine rich protein	fenebrutinib	5.56E-05
P25705	ATP synthase subunit alpha, mitochondrial	fenebrutinib	5.60E-05

Q9Y5H3	Protocadherin gamma-A10	PF-05190457	5.66E-05
Q9NU63	Zinc finger protein 57 homolog	fenebrutinib	5.67E-05
P43363	Melanoma-associated antigen 10	AI-10-49	5.87E-05
Q13469	Nuclear factor of activated T-cells, cytoplasmic 2	AI-10-49	5.91E-05
Q8NG77	Olfactory receptor 2T12	AI-10-49	5.91E-05
Q9Y2G8	DnaJ homolog subfamily C member 16	PF-05190457	5.91E-05
Q9ULV8	E3 ubiquitin-protein ligase CBL-C	fenebrutinib	5.93E-05
Q96EF0	Myotubularin-related protein 8	elbasvir	6.13E-05
Q8TD16	Protein bicaudal D homolog 2	abemaciclib	6.22E-05
P49459	Ubiquitin-conjugating enzyme E2 A	abemaciclib	6.22E-05
Q6XZF7	Dynamin-binding protein	AI-10-49	6.25E-05
Q9Y4I1	Unconventional myosin-Va	AI-10-49	6.25E-05
Q96FL9	Polypeptide N-acetylgalactosaminyltransferase 14	MK-5046	6.37E-05
Q9HD67	Unconventional myosin-X	MK-5046	6.37E-05
Q13214	Semaphorin-3B	MK-5046	6.41E-05
Q53H47	Histone-lysine N-methyltransferase SETMAR	MK-5046	6.41E-05
P46776	60S ribosomal protein L27a	AI-10-49	6.45E-05
Q15306	Interferon regulatory factor 4	AI-10-49	6.45E-05
Q2TAA2	Isoamyl acetate-hydrolyzing esterase 1 homolog	abemaciclib	6.54E-05
O43324	Eukaryotic translation elongation factor 1 epsilon-1	PF-05190457	6.63E-05
Q96R27	Olfactory receptor 2M4	fenebrutinib	6.64E-05
Q5VXU1	Sodium/potassium-transporting ATPase subunit beta-1-interacting protein 2	fenebrutinib	6.64E-05
Q5VWQ8	Disabled homolog 2-interacting protein	fenebrutinib	6.75E-05
Q9UJF2	Ras GTPase-activating protein nGAP	elbasvir	6.80E-05
O75923	Dysferlin	fenebrutinib	6.82E-05
O75592	E3 ubiquitin-protein ligase MYCBP2	PF-05190457	6.88E-05
Q14714	Sarcospan	CFI-402257	6.88E-05
Q12798	Centrin-1	CFI-402257	6.88E-05
Q9H9Z2	Protein lin-28 homolog A	fenebrutinib	7.08E-05
Q8N2A8	Mitochondrial cardiolipin hydrolase	fenebrutinib	7.08E-05
Q5VV41	Rho guanine nucleotide exchange factor 16	abemaciclib	7.10E-05
Q96QT6	PHD finger protein 12	fenebrutinib	7.13E-05
Q13948	Protein CASP	AI-10-49	7.18E-05
Q9NXH9	tRNA	AI-10-49	7.18E-05
Q969E2	Secretory carrier-associated membrane protein 4	fenebrutinib	7.24E-05
P58107	Epiplakin	fenebrutinib	7.24E-05
P55036	26S proteasome non-ATPase regulatory subunit 4	NMS-P715	7.39E-05

Q11128	4-galactosyl-N-acetylglucosaminide 3-alpha-L-fucosyltransferase FUT5	AI-10-49	7.44E-05
Q86W28	NACHT, LRR and PYD domains-containing protein 8	AI-10-49	7.45E-05
O43566	Regulator of G-protein signaling 14	Q-203	7.62E-05
Q9BVI0	PHD finger protein 20	Q-203	7.62E-05
Q93074	Mediator of RNA polymerase II transcription subunit 12	PF-05190457	7.69E-05
P51530	DNA replication ATP-dependent helicase/nuclease DNA2	PF-05190457	7.69E-05
Q6NS38	DNA oxidative demethylase ALKBH2	AI-10-49	7.76E-05
B4DJY2	Transmembrane protein 233	AI-10-49	7.78E-05
Q8N807	Protein disulfide-isomerase-like protein of the testis	PF-05190457	7.81E-05
P13929	Beta-enolase	AI-10-49	7.82E-05
Q9NRJ7	Protocadherin beta-16	AI-10-49	7.82E-05
O95751	Protein LDOC1	MK-5046	7.86E-05
Q92947	Glutaryl-CoA dehydrogenase, mitochondrial	fenebrutinib	7.90E-05
Q96J94	Piwi-like protein 1	fenebrutinib	7.99E-05
O15400	Syntaxin-7	adapalene	8.10E-05
O00192	Armadillo repeat protein deleted in velo-cardio-facial syndrome	adapalene	8.10E-05
Q17RD7	Synaptotagmin-16	CFI-402257	8.20E-05
A0PJZ3	Glucoside xylosyltransferase 2	MK-5046	8.35E-05
Q53GG5	PDZ and LIM domain protein 3	PF-05190457	8.48E-05
P20929	Nebulin	PF-05190457	8.48E-05
Q5NDL2	EGF domain-specific O-linked N-acetylglucosamine transferase	abemaciclib	8.55E-05
Q10570	Cleavage and polyadenylation specificity factor subunit 1	fenebrutinib	8.65E-05
Q53FE4	Uncharacterized protein C4orf17	AI-10-49	8.74E-05
Q96T21	Selenocysteine insertion sequence-binding protein 2	cilofexor	8.74E-05
P17980	26S proteasome regulatory subunit 6A	PF-05190457	8.76E-05
Q8TE77	Protein phosphatase Slingshot homolog 3	tropifexor	8.80E-05
Q96LD4	E3 ubiquitin-protein ligase TRIM47	tropifexor	8.80E-05
P25205	DNA replication licensing factor MCM3	fenebrutinib	8.82E-05
Q9NQC7	Ubiquitin carboxyl-terminal hydrolase CYLD	fenebrutinib	8.82E-05
Q71F23	Centromere protein U	NMS-P715	9.02E-05
P46778	60S ribosomal protein L21	fenebrutinib	9.25E-05
Q04727	Transducin-like enhancer protein 4	AI-10-49	9.30E-05
O14556	Glyceraldehyde-3-phosphate dehydrogenase, testis-specific	AI-10-49	9.30E-05
Q92994	Transcription factor IIIB 90 kDa subunit	fenebrutinib	9.34E-05

Q15646	2'-5'-oligoadenylate synthase-like protein	fenebrutinib	9.34E-05
O15131	Importin subunit alpha-6	fenebrutinib	9.55E-05
Q14254	Flotillin-2	fenebrutinib	9.55E-05
O75764	Transcription elongation factor A protein 3	CFI-402257	1.00E-04
P40425	Pre-B-cell leukemia transcription factor 2	MK-5046	1.01E-04
O43766	Lipoyl synthase, mitochondrial	PF-05190457	1.02E-04
Q969L2	Protein MAL2	fenebrutinib	1.03E-04
Q9NSG2	Uncharacterized protein C1orf112	CCT137690	1.03E-04
Q9H892	Tetratricopeptide repeat protein 12	PF-05190457	1.04E-04
O95248	Myotubularin-related protein 5	CCT137690	1.04E-04
O60481	Zinc finger protein ZIC 3	PF-05190457	1.06E-04
Q9UBK8	Methionine synthase reductase	AI-10-49	1.07E-04
Q4G0J3	La-related protein 7	CCT137690	1.07E-04
Q8WW32	High mobility group protein B4	CCT137690	1.09E-04
P23297	Protein S100-A1	MK-5046	1.11E-04
Q8TCB7	tRNA N	elbasvir	1.12E-04
Q8IXH6	Tumor protein p53-inducible nuclear protein 2	abemaciclib	1.13E-04

Table D in S1 Text: Predicted ligands for the transcription factors and transcription activity related proteins. * labels the transcription factors.

Uniprot ID	Protein name	Drug name	e-value
P22670	MHC class II regulatory factor RFX1	PF-05190457	8.66E-06
P40425	Pre-B-cell leukemia transcription factor 2	MK-5046	1.01E-04
Q7RTS3	Pancreas transcription factor 1 subunit alpha	MK-5046	9.50E-06
Q13469	T-cell transcription factor NFAT1	AI-10-49	5.91E-05
Q92754	Transcription factor AP-2 gamma	IACS-10759	3.26E-05
Q92994	Transcription factor IIIB 90 kDa subunit	Fenebrutinib	9.34E-05
Q93074	Mediator of RNA polymerase II transcription subunit 12	PF-05190457	7.69E-05
Q9BVI0	Transcription factor TZP	Q-203	7.62E-05
Q9BYE0	Transcription factor HES-7	MK-5046	4.91E-05
D6RGH6	Multicilin	Fenebrutinib	5.84E-06
O60481	Zinc finger protein ZIC 3	PF-05190457	1.06E-04
O95231	VENT-like homeobox protein 2	NMS-P715	1.66E-05
O95409	Zinc finger protein ZIC 2	Fenebrutinib	2.03E-05
P05455	Sjoegren syndrome type B antigen	AI-10-49	3.80E-05
P10073	Zinc finger and SCAN domain-containing protein 22	PF-05190457	5.22E-05
P42568	mixed-lineage leukemia translocated to chromosome 3 protein	AI-10-49	7.54E-06
P49640	Homeobox even-skipped homolog protein 1	CFI-402257	8.56E-06
P56179	Homeobox protein DLX-6	Fenebrutinib	2.05E-05
P61371	Insulin gene enhancer protein ISL-1	Fenebrutinib	3.23E-05
Q02575	Nescient helix loop helix 1	AI-10-49	8.47E-06
Q04727	Transducin-like enhancer protein 4	AI-10-49	9.30E-05
Q13948	Protein CASP	Tropifexor	4.99E-05
Q13948	Protein CASP	AI-10-49	7.18E-05
Q15306	Interferon regulatory factor 4	AI-10-49	6.45E-05
Q6NSZ9	Zinc finger and SCAN domain-containing protein 25	Fenebrutinib	3.23E-05
Q8WW32	High mobility group protein B4	PF-05190457	5.54E-05
Q8WW32	High mobility group protein B4	CCT137690	1.09E-04
Q96KN3	Homeobox protein PKNOX2	Abemaciclib	3.86E-05
Q96QT6	PHD finger protein 12	Fenebrutinib	7.13E-05
Q9C019	Tripartite motif-containing protein 15	Abemaciclib	5.08E-05
Q9C035	Tripartite motif-containing protein 5	AI-10-49	1.15E-05
Q9H7E2	Tudor domain-containing protein 3	NMS-1286937	2.86E-05
Q9HBT7	Zinc finger protein 287	CCT137690	2.22E-06
Q9NU63	Zinc finger protein 57 homolog	Fenebrutinib	5.67E-05
Q9UBS8	E3 ubiquitin-protein ligase RNF14	PF-05190457	4.56E-05
Q9UC06	Zinc finger protein 70	Fenebrutinib	1.06E-06
Q9UIE0	Zinc finger protein 230	CFI-402257	1.39E-05
Q9UJQ4	Sal-like protein 4	MK-5046	4.26E-05
Q9UKY1	Zinc fingers and homeoboxes protein 1	CFI-402257	3.20E-05

Table E in S1 Text: Chemicals interacted with undruggable human proteins

Drug name	Clinical phase	Mechanism of Action	Number of targeted proteins
AI-10-49	Preclinical	core binding factor inhibitor	63
Fenebrutinib	Phase 2	Bruton's tyrosine kinase (BTK) inhibitor	56
PF-05190457	Phase 2	growth hormone secretagogue receptor inverse agonist	42
Abemaciclib	Launched	CDK inhibitor	21
MK-5046	Preclinical	bombesin receptor agonist	18
CFI-402257	Phase 1/Phase 2	dual specificity protein kinase inhibitor	14
CCT137690	Preclinical	Aurora kinase inhibitor	11
Tropifexor	Phase 2	FXR agonist	5
NMS-1286937	Phase 2	PLK inhibitor	5
NMS-P715	Preclinical	protein kinase inhibitor	5
Elbasvir	Launched	HCV inhibitor	5
Cilofexor	Phase 3	FXR agonist	4
CDK9-IN-6	Preclinical	CDK inhibitor	3
piperaquine-phosphate	Launched	antimalarial agent	3
Q-203	Phase 2	ATP synthase inhibitor	3
ABT 702 dihydrochloride	Preclinical	adenosine kinase inhibitor	2
Ziritaxestat	Phase 3	autotaxin inhibitor	2
Adapalene	Launched	retinoid receptor agonist	1
Acalabrutinib	Launched	Bruton's tyrosine kinase (BTK) inhibitor	1
IACS-10759 Hydrochloride	Preclinical	mitochondrial complex I inhibitor	1
NVP-CGM097	Phase 1	MDM inhibitor	1

Table F in S1 Text: Targets predicted by PortalCG for AI-10-49, fenebrutinib, PF-05190457 and their docking score from Autodock Vina.

Target protein	Drug name	Autodock score (kcal/mol)	PortalCG e-value
Q92673	AI-10-49	-7.3	5.43E-06
P42568	AI-10-49	-6.8	7.54E-06
O75689	AI-10-49	-5.6	1.51E-05
Q86XR7	AI-10-49	-10.1	2.15E-05
Q5RKV6	AI-10-49	-7.7	3.31E-05
Q13469	AI-10-49	-8.6	5.91E-05
O14556	AI-10-49	-9.8	9.30E-05
Q9UBK8	AI-10-49	-8.2	1.07E-04
Q9NQX4	Fenebrutinib	-9.5	1.55E-05
O75923	Fenebrutinib	-8.1	6.82E-05
Q9H9Z2	Fenebrutinib	-8.2	7.08E-05
Q96J94	Fenebrutinib	-8.2	7.99E-05
Q9NQC7	Fenebrutinib	-8.3	8.82E-05
Q9UKR8	PF-05190457	-8.3	2.38E-06
P17735	PF-05190457	-7.3	1.03E-05
O75886	PF-05190457	-8.4	2.50E-05
Q9NZM3	PF-05190457	-7.6	3.69E-05
P08118	PF-05190457	-7.9	5.33E-05
P57729	PF-05190457	-8.6	5.54E-05
P51530	PF-05190457	-9.4	7.69E-05
Q53GG5	PF-05190457	-7.7	8.48E-05

Table G in S1 Text: Functional Annotation enrichment for human proteins in Tbio selected by PortalCG

David Functional Annotation enrichment analysis				
Enriched terms in UniProtKB keywords	Number of proteins involved	Percentage of proteins involved	P-value	Modified Benjamini p-value
Alternative splicing	153	69.9	2.70E-08	6.80E-06
Phosphoprotein	126	57.5	1.70E-07	2.20E-05
Cytoplasm	82	37.4	3.20E-06	2.40E-04
Nucleus	87	39.7	3.70E-06	2.40E-04
Metal-binding	61	27.9	2.00E-04	1.00E-02

Table H in S1 Text: Chemicals interacted with human proteins in Tbio

rug name	Clinical phase	Mechanism of Action	Number of targeted proteins
AI-10-49	Preclinical	core binding factor inhibitor	52
fenebrutinib	Phase 2	Bruton's tyrosine kinase (BTK) inhibitor	45
PF-05190457	Phase 2	growth hormone secretagogue receptor inverse agonist	36
abemaciclib	Launched	CDK inhibitor	20
MK-5046	Preclinical	bombesin receptor agonist	15
CFI-402257	Phase 1/Phase 2	dual specificity protein kinase inhibitor	14
CCT137690	Preclinical	Aurora kinase inhibitor	7
NMS-1286937	Phase 2	PLK inhibitor	5
tropifexor	Phase 2	FXR agonist	4
NMS-P715	Preclinical	protein kinase inhibitor	4
cilofexor	Phase 3	FXR agonist	3
CDK9-IN-6	Preclinical	CDK inhibitor	3
piperaquine-phosphate	Launched	antimalarial agent	3
elbasvir	Launched	HCV inhibitor	3
ABT-702	Preclinical	adenosine kinase inhibitor	2
adapalene	Launched	retinoid receptor agonist	2
Q-203	Phase 2	ATP synthase inhibitor	2
ziritaxestat	Phase 3	autotaxin inhibitor	2
acalabrutinib	Launched	Bruton's tyrosine kinase (BTK) inhibitor	1
IACS-10759	Preclinical	mitochondrial complex I inhibitor	1
CGM097	Phase 1	MDM inhibitor	1

Table I in S1 Text: Functional Annotation enrichment for undruggable human disease proteins without Tbio selected by PortalCG

David Functional Annotation enrichment analysis				
Enriched terms in UniProtKB keyword	Number of proteins involved	Percentage of proteins involved (%)	P-value	Modified Benjamini p-value
Zinc-finger	80	23	7.60E-16	1.20E-13
Zinc	85	24.4	1.20E-11	9.90E-10
Metal-binding	96	27.6	4.30E-06	2.30E-04
DNA-binding	62	17.8	7.90E-06	3.20E-04
Transcription regulation	61	17.5	5.60E-04	1.80E-02
Transcription	61	17.5	1.10E-03	3.00E-02

Table J in S1 Text: Top ranked diseases associated with undruggable human proteins excluding Tbio selected by PortalCG

Disease Name	# of undruggable proteins associated with the disease
Body Height	31
Colorectal Carcinoma	28
Malignant neoplasm of breast	26
Breast Carcinoma	18
Blood Protein Measurement	18
Leukemia, Myelocytic, Acute	17
Carcinogenesis	17
Neoplasm Metastasis	17
Liver carcinoma	15
Malignant neoplasm of prostate	15

Table K in S1 Text: Chemicals interacted with undruggable human proteins excluding Tbio

Drug_name	Clinical phase	Mechanism of Action	Number of targeted proteins
fenebrutinib	Phase 2	Bruton's tyrosine kinase (BTK) inhibitor	80
PF-05190457	Phase 2	growth hormone secretagogue receptor inverse agonist	50
MK-5046	Preclinical	bombesin receptor agonist	38
CCT137690	Preclinical	Aurora kinase inhibitor	36
AI-10-49	Preclinical	core binding factor inhibitor	31
abemaciclib	Launched	CDK inhibitor	26
CFI-402257	Phase 1/Phase 2	dual specificity protein kinase inhibitor	20
NMS-P715	Preclinical	protein kinase inhibitor	14
NMS-1286937	Phase 2	PLK inhibitor	11
elbasvir	Launched	HCV inhibitor	8
cilofexor	Phase 3	FXR agonist	7
ABBV-744	Phase 1	bromodomain inhibitor	7
tropifexor	Phase 2	FXR agonist	7
CDK9-IN-6	Preclinical	CDK inhibitor	6
adapalene	Launched	retinoid receptor agonist	4
Q-203	Phase 2	ATP synthase inhibitor	4
PLX8394	Phase 1/Phase 2	serine/threonine kinase inhibitor	4
ABT-702	Preclinical	adenosine kinase inhibitor	4
NVP-BSK805	Preclinical	JAK inhibitor	3
OTS167	Phase 1/Phase 2	maternal embryonic leucine zipper kinase inhibitor	3
CHIR-99021	Preclinical	glycogen synthase kinase inhibitor	3
DBPR-211	Preclinical	cannabinoid receptor antagonist	2
A-887826	Preclinical	sodium channel blocker	2
integrin-antagonist-1	Phase 1	integrin antagonist	2
piperaquine-phosphate	Launched	antimalarial agent	2
cenerimod	Phase 2	sphingosine 1-phosphate receptor modulator	1
peposertib	Phase 1/Phase 2	DNA dependent protein kinase inhibitor	1
tezacaftor	Launched	CFTR channel agonist	1
cot-inhibitor-2	Preclinical	MAPK-interacting kinase inhibitor	1
itacitinib	Phase 3	JAK inhibitor	1
10-hydroxycamptothecin	Preclinical	topoisomerase inhibitor	1
alectinib	Launched	ALK tyrosine kinase receptor inhibitor	1
adarotene	Phase 1	retinoid receptor agonist	1
acalabrutinib	Launched	Bruton's tyrosine kinase (BTK) inhibitor	1
XL041	Preclinical	LXR agonist	1
WAY-207024	Preclinical	gonadotropin releasing factor hormone receptor antagonist	1
MK-5108	Phase 1	Aurora kinase inhibitor	1
CGM097	Phase 1	MDM inhibitor	1
CD-437	Preclinical	retinoid receptor agonist	1
AMG-925	Phase 1	CDK inhibitor|FLT3 inhibitor	1
ACT-132577	Launched	endothelin receptor antagonist	1
ziritaxestat	Phase 3	autotaxin inhibitor	1

3 Additional figures

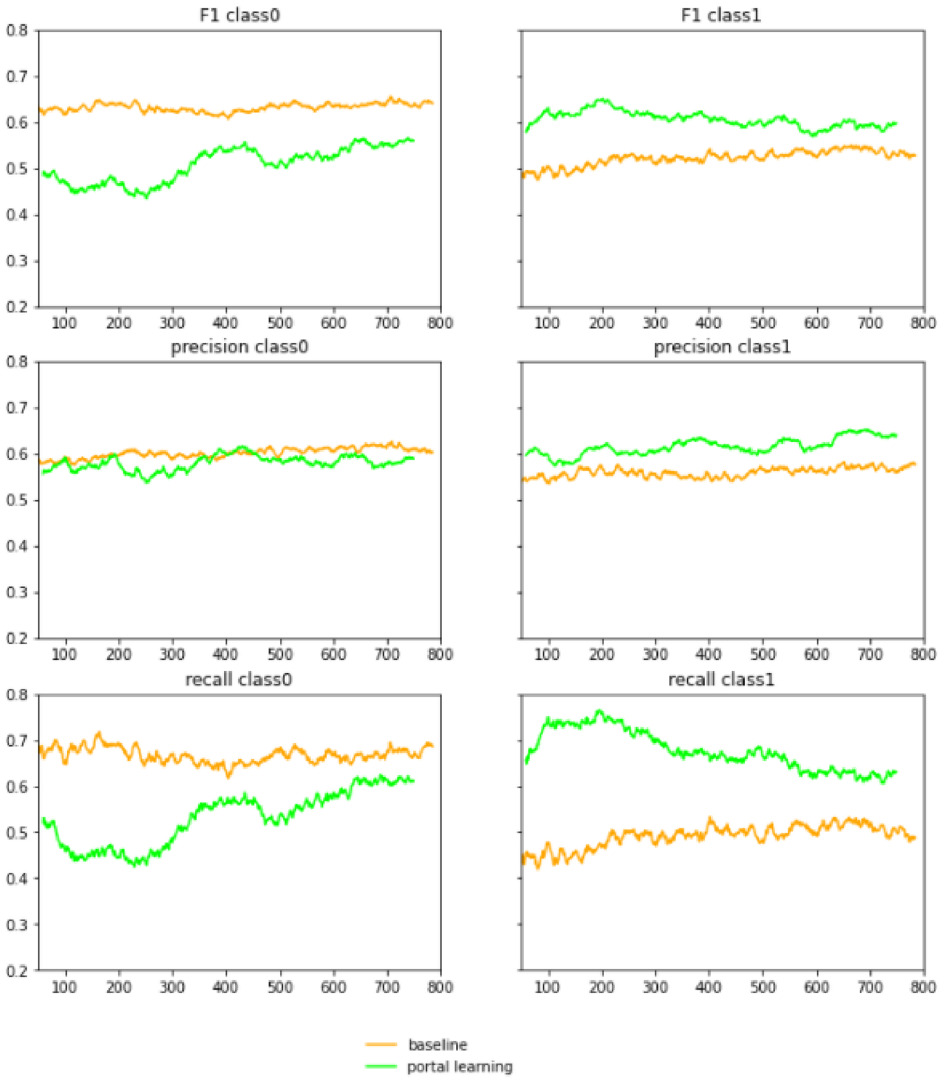

Fig A in S1 Text: In the main text, overall evaluation across positive and negative classes are reported, such as F1, ROC-AUC, PR-AUC. Here is a breakdown of performance in each class, where class0 is negative, i.e. not binding, class1 is positive, i.e. binding. against DISAE as baseline

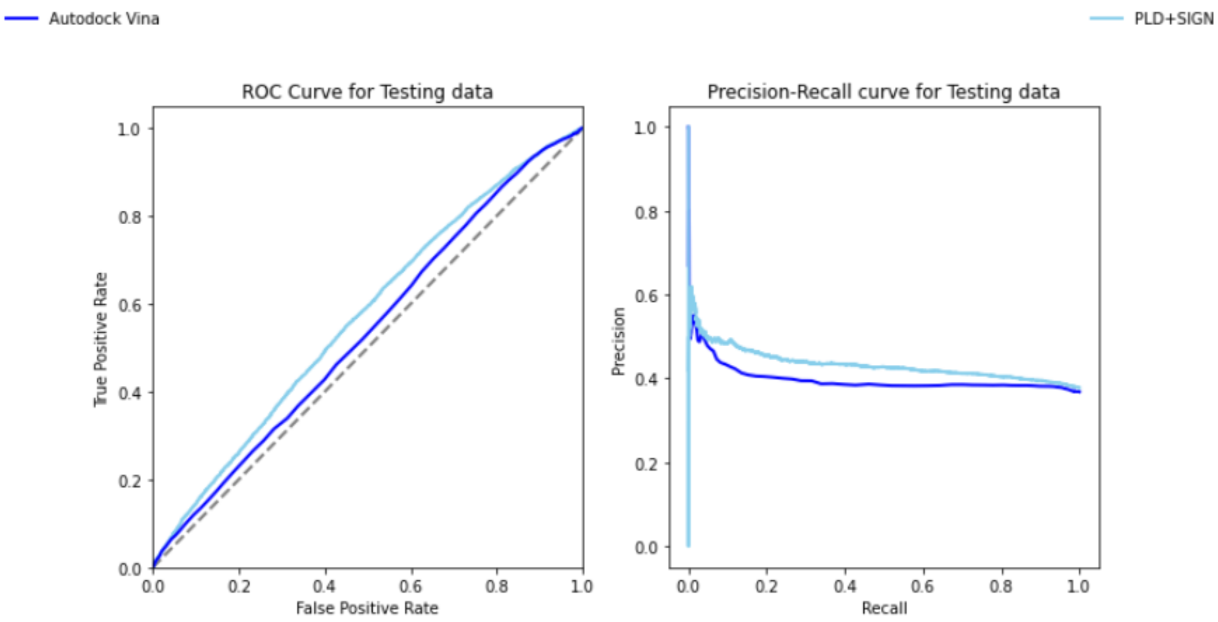

Fig B in S1 Text: Performance comparison of PLD+SIGN and Autodock Vina using the same OOD-test set as in main text Figure 3: ROC-AUCs of Autodock Vina and PLD+SIGN are 0.535 and 0.569, respectively. PR-AUCs of Autodock Vina and PLD+SIGN 0.398 and 0.433, respectively.

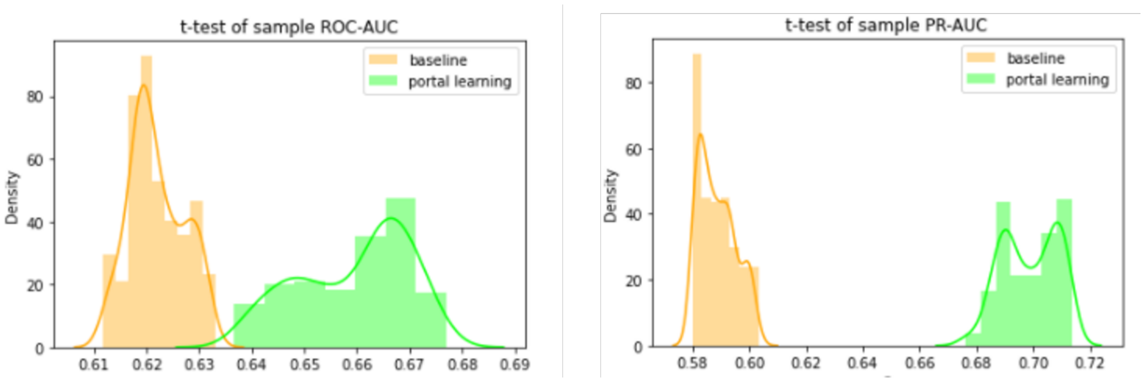

Fig C in S1 Text: t-test comparison. The p-values for both ROC-AUC and PR-AUC are close to 0 against DISAE as baseline

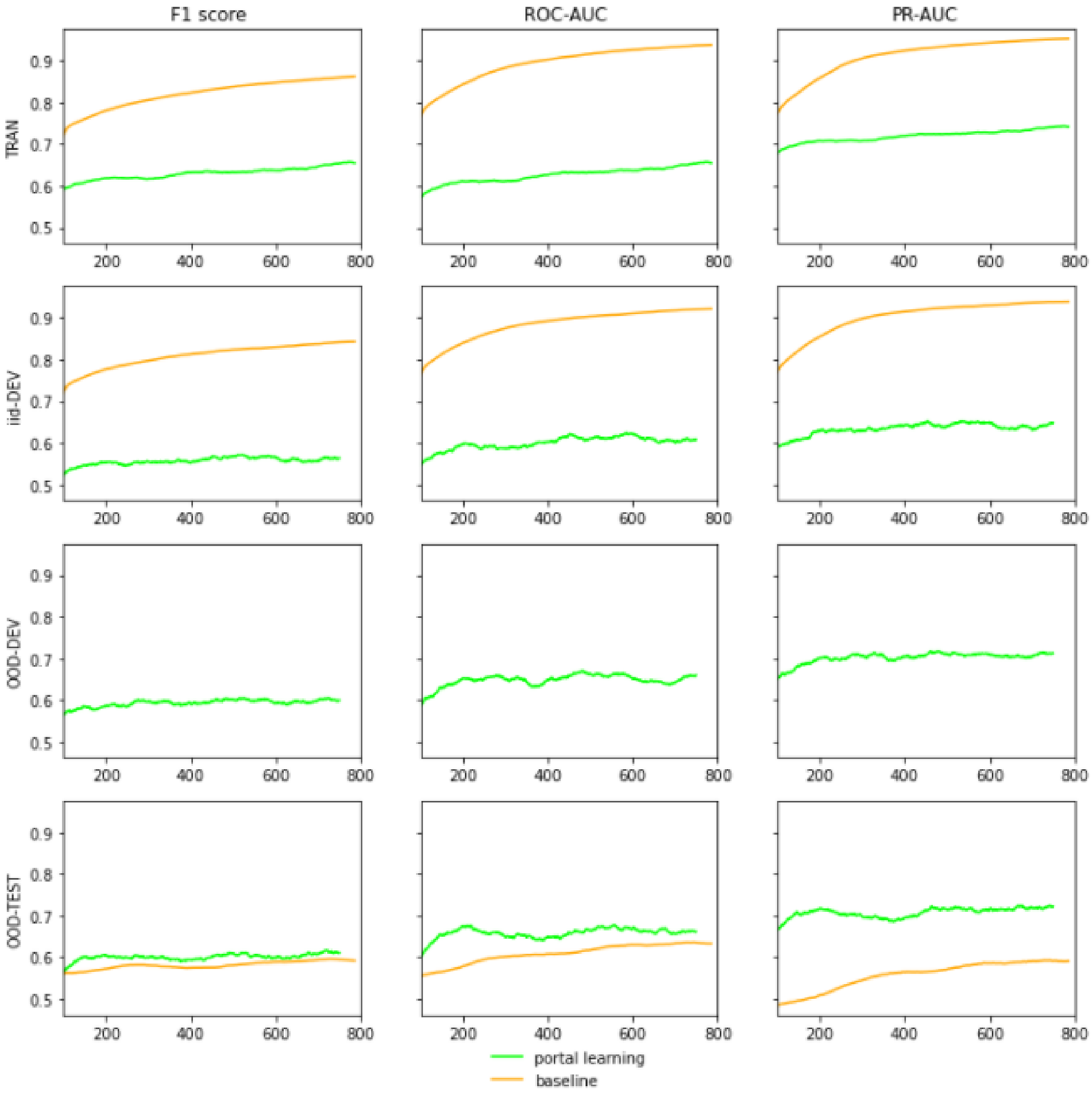

Fig D in S1 Text: Stress model selection performance curves against DISAE as baseline

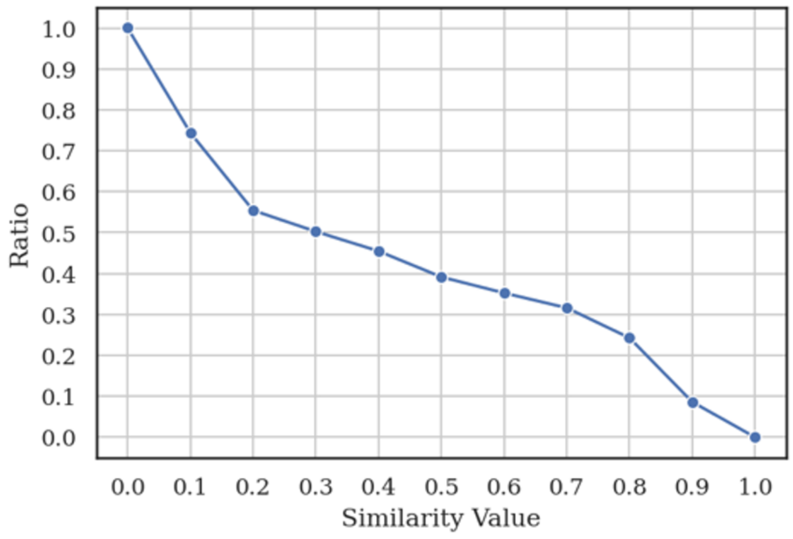

Fig E in S1 Text: The ratio of inactive CPIs vs active CPIs under different Tanimoto coefficients of chemical similarities in the training data. The ratio of total inactive CPIs vs active CPIs is 1:1.

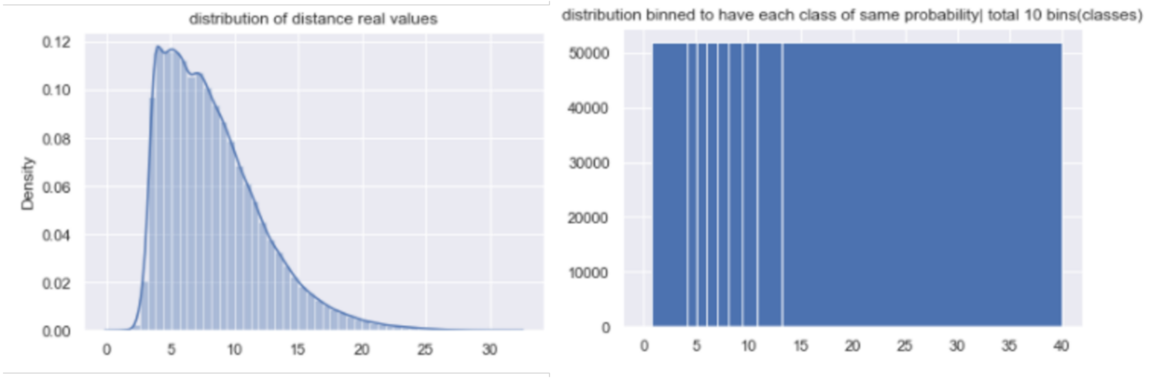

Fig F in S1 Text: Histogram equalization results: the left panel shows the original distribution of distance real values; to formalize a multi-class classification where each class has equal probability, histogram equalization transforms the distribution to the right panel of 10 bins, each as a class.

References

[1] O. Trott and A. J. Olson, “Autodock vina: improving the speed and accuracy of docking with a new scoring function, efficient optimization and multithreading,” *Journal of Computational Chemistry*, vol. 31, pp. 455–461, 2010.

[2] S. Madapa, S. Gadhiya, T. Kurtzman, I. L. Alberts, S. Ramsey, M. Reith, and W. W. Harding, “Synthesis and evaluation of c9 alkoxy analogues of (-)-stepholidine as dopamine receptor ligands,” *European journal of medicinal chemistry*, vol. 125, pp. 255–268, 2017.

[3] R. K. Pal, S. Gadhiya, S. Ramsey, P. Cordone, L. Wickstrom, W. W. Harding, T. Kurtzman, and E. Gallicchio, “Inclusion of enclosed hydration effects in the binding free energy estimation of dopamine d3 receptor complexes,” *Plos one*, vol. 14, no. 9, p. e0222902, 2019.

[4] S. Gadhiya, P. Cordone, R. K. Pal, E. Gallicchio, L. Wickstrom, T. Kurtzman, S. Ramsey, and W. W. Harding, “New dopamine d3-selective receptor ligands containing a 6-methoxy-1, 2, 3, 4-tetrahydroisoquinolin-7-ol motif,” *ACS medicinal chemistry letters*, vol. 9, no. 10, pp. 990–995, 2018.

[5] P. Cordone, H. K. Namballa, B. Muniz, R. K. Pal, E. Gallicchio, and W. W. Harding, “New tetrahydroisoquinoline-based d3r ligands with an o-xylenyl linker motif,” *Bioorganic & Medicinal Chemistry Letters*, vol. 42, p. 128047, 2021.
